# Gut microbiome of *Vespa orientalis*: functional insights and potential honey bee pathogen dynamics

**DOI:** 10.1186/s42523-025-00460-6

**Published:** 2025-09-30

**Authors:** Simone Cutajar, Chiara Braglia, Daniele Alberoni, Martina Mifsud, Loredana Baffoni, Jorge Spiteri, Diana Di Gioia, David Mifsud

**Affiliations:** 1 Dipartimento di Scienze e Tecnologie Agro-Alimentari (DISTAL), University of Bologna, Viale Fanin 42, Bologna, 40127 Italy; 2 Institute of Earth Systems, L-Università tà Malta, Msida, Malta; 3 Malta Beekeepers’ Association VO1527 c/o, Volunteer Centre, 181, Triq Melita, Valletta, 1129 VLT Malta

**Keywords:** Pollinators, Pathogens, Functional prediction, *Nosema ceranae*, *Crithidia bombi*, *Spiroplasma*, *Arsenophonus*

## Abstract

*Vespa orientalis*, the oriental hornet, is an emerging predator of honey bees whose ecological impact and microbial ecology remain poorly understood. Here, we present the first detailed characterisation of its gut microbiota by integrating 16S rRNA gene sequencing, predicted microbial function, pathogen screening, and a three-year beekeeper survey across urban and rural sites in Malta. Hornets were sampled from four locations and classified by observed foraging behaviour, either predation on honey bees or scavenging on cat food.

Survey data confirmed consistent *V. orientalis* sightings and seasonal colony losses, particularly during peak foraging months. Microbiome analysis revealed a conserved core community dominated by *Spiroplasma*, *Arsenophonus*, and *Rosenbergiella*, with overall diversity stable across sites and diets. However, specific taxa varied with foraging behaviour. For example, *Arsenophonus* was enriched in bee-predating hornets, while *Enterobacter* and *Serratia* were more common in scavenging individuals, suggesting environmental and dietary influences on microbiota composition. Predicted functional profiles remained broadly conserved, reflecting robust nutrient metabolism and potential detoxification capabilities, with some variations related to the diet behaviour.

Pathogen screening detected *Nosema ceranae* and *Crithidia bombi* in a substantial proportion of hornets, including those not observed feeding on bees. Although our findings do not demonstrate pathogen transmission, they support the hypothesis that *V. orientalis* may act as a transient carrier, potentially contributing to pathogen persistence via environmental exposure.

Together, these results reveal the dietary flexibility and microbial flexibility within the gut microbiome of *V. orientalis*, and highlight its potential involvement in pollinator pathogen dynamics.

**Supplementary Information:**

The online version contains supplementary material available at 10.1186/s42523-025-00460-6.

## Introduction

*Vespa orientalis*, the oriental hornet, is a large eusocial insect belonging to the Vespidae family, native to the southeastern Mediterranean region, including Malta, southern Italy, Cyprus, as well as northeastern and eastern Africa, the Middle East, and Central Asia [1–3]. Its expanding presence, via accidental human-assisted introductions and dispersal [4], in new regions such as Spain [5, 6], Romania [7], France [8], north Italy [9, 10], the Greek islands [11], South America (Brazil and Chile) [12] and North America (Mexico) [13] has raised concern over its invasive potential, attributed to traits such as adaptability to urban and peri-urban environments [4, 10] and diverse foraging behaviour, including scavenging on protein-rich and sugar-rich materials [4, 10, 14].

Known for establishing underground nests and preying on honey bees and other insects, *V. orientalis* has become increasingly problematic for apiculture, with reports of colony losses in its native range, including Malta [15] (Appendix 1). Whether these losses arise from direct predation, stress-induced vulnerability, or pathogen transfer remains an open question [4, 10, 14]. In addition to threatening apiculture, it was recently suggested by Zucca et al. [16] that *V. orientalis* may also pose indirect public health risks due to its scavenging behaviour in urban environments, which can expose it to pathogens of human relevance [16].

Recent studies have highlighted the fundamental role of the gut microbiome in social insects, influencing host health, digestion functionality, immune function, behaviour and adaptability [17, 18]. In hornets, microbiome studies on *V. mandarina*, *V. simillima*, and *V. velutina* (including its subspecies *V. velutina nigritorax*) show communities dominated by Bacillota (comprising the reclassified Mycoplasmatota), Actinomycetota and Pseudomonadota [19–21]. These communities are shaped by caste, developmental stage, and geography, and in some cases include honey bee-associated bacteria as discussed by Cini et al. [22]. Despite the hornet’s growing prevalence and ecological impact in both its native and invaded ranges, the gut microbiome of *V. orientalis* has yet to be characterised. Establishing a baseline community profile would clarify host-microbe relationships, reveal whether the hornet picks up pollinator-associated pathogens, and lay the groundwork for assessing predator-prey or flower-mediated microbial exchange. Moreover, given the invasive potential of *V. orientalis* and its increasing impact on honey bee populations, characterising its microbial community may offer important insights into pathogen dynamics and broader ecological consequences [16, 23]. This study presents the first comprehensive characterisation of the *V. orientalis* gut microbiome and its predicted functionality, combining high-throughput sequencing and targeted detection of pathogen screening. By analysing hornets from urban and natural sites in Malta, where they either prey on honey bees or scavenge leftover anthropogenic protein sources (such as leftover cat food), we explore how diet and environment shape microbial composition and feed-related functionality. In addition to microbiome profiling, we incorporated a beekeeper survey to contextualise the study within the Maltese apicultural landscape. The survey shows Maltese beekeepers’ growing concern of the impact of *V. orientalis* on honey bee colonies in Malta and underscores the need to explore all possible ecological interactions. Given the overlap between *V. orientalis* activity and seasonal colony losses, and the growing recognition that predator-prey interactions may facilitate microbial exchange, this study also explored the potential for pathogen carriage by hornets, without assuming a causal link to colony decline. This integrative approach allowed us to examine how foraging behaviour and site-level variation shape the hornet gut microbiome and its possible role in pathogen ecology.

Building on evidence from other social insects [24, 25] our findings raise the possibility of microbial exchange between *V. orientalis* and its ecological contacts. However, confirming this will require future studies that analyse hornet, prey and environmental microbiota in parallel, at the same sites and time points. While our study does not establish causality or transmission dynamics, the microbiome patterns we elucidate offer a preliminary step toward understanding the species’ ecological adaptability and may inform future research on pathogen surveillance or alternative management strategies.

## Materials and methods

### Beekeeper survey data collection

To assess the perceived impact of *V. orientalis* on beekeeping in Malta, survey data were collected by the Malta Beekeeping Association (MBKA, VO 1527) from 2022 to 2024. The surveys aimed to document beekeeper-reported observations, colony and brood losses, mitigation efforts, and the effectiveness of control measures against *V. orientalis*. The dataset was compiled from voluntary responses by MBKA members, representing a longitudinal perspective on their self-reported perception of the species’ effects on apiculture in Malta. The survey provides insights into yearly variations in perceived apicultural impact of *V. orientalis* and beekeeper interventions. Each annual survey contained a structured set of questions designed to capture key trends, such as reported colony losses attributed to hornet presence, and beekeeper interventions to mitigate the threat. While the questionnaire evolved slightly over the three-year period, core questions remained consistent to allow for comparative analysis. The questionnaire opened with a coloured photograph of *V. orientalis* to ensure accurate species recognition. Because *V. orientalis* is currently the only known hornet species recorded in Malta [14], and all respondents were experienced MBKA beekeepers, the risk of misidentification was considered negligible. The data collected, are self-reported perceptions of colony loss induced by *V. orientalis* presence and the results should be interpreted accordingly. The responses were compiled, anonymised, and provided by the MBKA for integration into this study. The surveys were conducted in Maltese and subsequently translated into English by the study authors. Survey results were analysed to detect changes in perceived hornet impact over time and assess the effectiveness of control measures.

Survey responses were aggregated by year (2022, 2023 and 2024), and a comparative analysis was performed to assess year-over-year trends in hornet sightings, reported colony losses, intervention rates, and perceived effectiveness of control measures. This analysis was descriptive and based on proportional comparisons across years, using the total number of respondents as the denominator for each year-specific metric (e.g., percentage of respondents reporting colony losses). No inferential statistics were applied due to limited and variable sample sizes across years.

### Samples collection

Adult *V. orientalis* female workers (Supplementary Figure S1 and Appendix 2) were collected from four site types categorised by dominant food source: ‘honey bee’ sites (locations with active apiaries) and ‘cat food’ sites (urban locations where hornets scavenged pet food). The sites from which *V. orientalis* individuals were sampled for honey bee predation were specifically selected based on beekeeper reports of confirmed hornet activity and honey bee colony losses. The authors observed active predation on honey bees during hornet sampling. Hornets in the ‘cat food’ group were observed feeding on processed cat food left outdoors for stray cats in urban and peri-urban areas. This protein source was consistently available across ‘cat food’ sites and hornets were frequently seen foraging on it during our collections. While we refer to this dietary category as ‘cat food’, we acknowledge that it likely serves as a proxy for broader anthropogenic protein sources accessible to hornets in human-modified environments. Other food scraps, such as discarded meat or processed foods, may also contribute to this dietary niche, but cat food was the only observed foraging substrate at the time of collection.

Sampling was conducted between September and October 2023 at four localities; an urban site in Imsida (VoU, University of Malta campus − 35°54’01"N 14°28’59"E), a peri-urban site in Qawra (VoQ − 35°56’50"N 14°25’19"E), a peri-urban site in San Ġwann (VoS − 35°54’26.6"N 14°27’49.3"E), and a natural site in Gudja (VoG − 35°51’18"N 14°30’35"E). In each site (Supplementary Figure S2), hornets were captured randomly using either a sweep net or an electric fly swatter, for a total of 70 samples. The handheld electric fly swatter delivers a very brief, high voltage, low current discharge that instantly immobilises the hornet without crushing or burning tissue or leaving any chemical residues. Specimens were placed on ice immediately after collection and transported to the University of Malta, where they were stored at -80 °C. They were subsequently shipped frozen to the University of Bologna for molecular analysis.

### DNA extraction

Prior to DNA extraction, adult hornets were superficially sterilised by rinsing in 70% ethanol for 30 sec and briefly air-dried, then dissected at controlled temperature near 0°C using ethanol-sterilised forceps. The last abdominal segment was carefully removed along with the entire gut and transferred into a sterile 1.5mL Eppendorf tube. A total of 70 gut samples were processed for microbial DNA extraction according to Baffoni et al. [26]. DNA was extracted using the PureLink™ Genomic DNA Mini Kit (Thermo Fisher Scientific), following the manufacturer’s protocol. The extracted DNA was quantified using a Qubit dsDNA HS Assay Broad Range Kit (Thermo Fisher Scientific).

### Quantitative polymerase chain reaction (qPCR)

The absolute quantification of bee pathogens (*Nosema ceranae*, *N. apis*, *N. bombi*, *Serratia*, *Crithidia bombi*, *C. mellificae*, *Lotmaria passim*, *Apicystis bombi*), human pathogens (*Listeria* and *Salmonella*), and total bacteria were quantified with specific primers listed in Supplementary Table S3. The PCR products for each target were purified with NucleoSpin^®^ Gel and PCR Clean-up (Macherey-Nagel), quantified with Qubit dsDNA Broad Range kit (Thermo Fisher Scientific) and converted in total amount of copies per microliter. Purified PCR products were used to generate standard curve-based quantification, obtained with serial dilution of the pre-amplified target amplicons (10^4^ to 10^8^ copies). Quantitative PCR protocols were carried out with QuantStudio^®^ 5 Real-Time PCR System (Applied Biosystems), according to Braglia et al. [27, 28]. Amplifications of each microbial target were carried out using PowerUp SYBR Green Master Mix (Applied Biosystems) in a final volume of 10µL. Absolute abundance of *N. ceranae* was corrected according to the 16S-like rRNA gene copy number according to Garrido et al. [29], whereas absolute abundance for *C. bombi* and *Serratia* (*luxS* gene) were not corrected because only one copy of the target gene is present per organism. Data were expressed as Log spores/gut for *Nosema*, Log cells/gut for *C. bombi*, Log *luxS* gene copies/gut for *Serratia*, and Log 16S rRNA copies/gut for total bacteria. qPCR analysis was performed on the same 70 individuals used for microbiome sequencing, with sample sizes per site as follows: Gudja (*n* = 28), Imsida (*n* = 24), San Ġwann (*n* = 15), and Qawra (*n* = 3).

### NGS library preparation and sequencing

Library preparation and sequencing were performed by IGA Technology Services S.r.l. (Udine, Italy). The V3-V4 hypervariable region of the bacterial 16S rRNA gene was amplified using the primer pair 341F (5′-CCTACGGGNGGCWGCAG-3′) and 785R (5′-GACTACHVGGGTATCTAATCC-3′). Sequencing was conducted on an Illumina NovaSeq platform, generating paired-end reads (2 × 250 bp). The sequencing targeted a depth of approximately 100,000 reads per sample, corresponding to 50,000 paired-end fragments. The sequencing service included PCR amplification, library preparation, and quality control (QC) checkpoints. Samples were directly processed through PCR amplification, and only successfully amplified libraries were used for sequencing. The sequencing platform was optimised to ensure a minimum of 95% of the target sequencing output (expressed in millions of reads).

### Bioinformatics

Raw sequencing reads were processed using QIIME2-amplicon-2024.2 [30] according to Fernandez De Landa et al. [31], with some minor modifications. Briefly, the paired-end reads were denoised, quality-filtered, and merged using the DADA2 plugin. Following merging and chimera removal, a rooted phylogenetic tree was constructed for diversity analysis. All samples were then rarefied to a uniform sequencing depth of 16,710 merged reads, which represented the lowest sequencing depth among the total 70 samples included in the final analysis. Since all samples met or exceeded this threshold, no samples were excluded during rarefaction. Taxonomic classification was performed using the Silva 138.1 database [32] with the plugin qiime feature-classifier using vsearch (classify-consensus-vsearch), employing a full-length sequence classifier. Prior to visualisation with qiime taxa barplot, taxonomic assignment and representative sequences were filtered to remove taxa annotated as “Mitochondria”, “Chloroplast”, and “Unassigned” using the plugin qiime taxa filter-seqs. Phylogenetic relationships were inferred by constructing a rooted tree using qiime phylogeny align-to-tree-mafft-fasttree plugin. Alpha and beta diversity analyses were conducted using this rooted tree and 16,710 as sampling-depth. Rarefaction curves were generated with qiime diversity plugin and the visualiser alpha-rarefaction (Supplementary Figure S3, S4 and S5). Relative abundance data were then processed using the qPCR total bacteria quantification for each sample (Sect. 2.5), and then normalised by the 16S copy number for each genus or species according to Raymann et al. [33], obtaining absolute abundance values for the *V. orientalis* gut microbiome.

### Identification of core microbiota and associated functions

To identify the core gut microbiota of *V. orientalis*, bacterial taxa consistently present across samples at a predefined prevalence threshold were considered. Microbial presence was assessed at the Amplicon Sequence Variant (ASV) level, and prevalence score (PS, %) was calculated as (*number of samples where the taxon is present total number of samples*) × 100. In this study, core gut microbiota was defined as taxa detected in at least 80% of samples (across all locations) with a relative abundance greater than 1%, following established thresholds [19, 34]. Gut core microbiome taxa genomes retrieved from the NCBI database and used for functionality study, are reported in Supplementary Table S1.

To assess the predicted functionality of the core gut microbiota, genomes of the representative bacterial strains were annotated with the RAST pipeline (SEED Viewer version 2.0) [35, 36] KEGG orthology database [37] and EggNOG [38]. Bacterial genomes were annotated and screened for complete Enzyme Commission (EC) numbers for gene function descriptions. All data were post-processed in R 4.3.3 (R Foundation for Statistical Computing; Vienna, Austria) to classify each gene into specific functional categories, using packages including dplyr, tidyr, readxl, tidyverse, httr, pheatmap, openxlsx, and ggplot2 for data visualisation.

Among the functional categories identified (fully reported in Supplementary Table S2), we focused on four groups that are most relevant not only to hornet nutrition, host-microbe interaction but also to the species’ adaptability and resilience in anthropogenic habitats:

(*i*) amino acids synthesis and fatty acid biosynthesis, to supply nutrients for larval food and cuticle formation, (*ii*) monosaccharides, polysaccharides, protein and alkaloids degradation, reflecting the mixed plant- and protein-based repertoire that hornets forage and scavenge on, (*iii*) nitrogen, vitamins, hormones and aromatic compounds metabolism, to help detoxify xenobiotics and correct vitamin imbalances common in urban environments, and (*iv*) resistance to antibiotics and toxic compounds, possible tolerance to antimicrobial residues and heavy metals. For each genome, the percentage distribution of genes across these categories was calculated. Only complete functional pathways were considered for this analysis. Therefore, to discriminate active pathways from incomplete ones, ECs were mapped to KEGG pathways using the KEGG REST API (https://www.kegg.jp/kegg/rest/). The resulting data were aggregated by microbial species, by sample, and by site to generate predicted pathway coverage profiles according to Alberoni et al. [39]. To provide a comparative estimation of the functional potential among microbial taxa across individuals, the functional absolute values were converted into relative values for each microbial taxon and expressed as ‘Predicted Score Value’ (ps). The ps value for each function was calculated as the relative abundance of each microbial taxon, based on the number of function-associated genes and normalised to the total predicted functional content of the sample. Predicted functional profiles were visualised as bubble plots, where bubble size represents the relative intensity of each function across samples or sampling sites. Moreover, pathways were visualised using bubble plots and clustered heatmaps, highlighting the top 20 most represented pathways across hornet samples and sites. Following this, a detailed assessment of the gut microbiota’s degradative potential in *V. orientalis* was conducted. The functional prediction focused on eight enzyme-/toxin-based traits that are mechanistically tied to *V. orientalis* biology. Specifically, chitin-, collagen- and general protease genes reflect the hornet’s need to digest insect cuticle and muscle, pectin- and hemicellulose-hydrolases capture the carbohydrate phase of the adult diet (nectar, honeydew, fruit juices), and putrescine-pathway genes track any possible nitrogen recycling. This analysis was conducted, to elucidate the possible impact of the different protein sources studied in this work (cat food and honey bees). Following Alberoni et al. [39], the number of enzymes in each functional category was normalised to the relative abundance of the corresponding core-gut genera. All values were log-transformed to facilitate visualisation.

### Statistical analysis

All statistical analyses were conducted using QIIME2-amplicon-2024.2 [30] and R version 4.3.3 to assess microbial diversity and composition across different food sources and geographic locations. Alpha diversity metrics, including Faith’s Phylogenetic Diversity, Observed Features, and Pielou’s Evenness, were calculated using the QIIME2 core-metrics-phylogenetic pipeline. Differences in alpha diversity between groups were assessed using the Kruskal-Wallis H test, a non-parametric method suitable for comparing microbial diversity across multiple independent groups [40], as implemented in the alpha-group-significance plugin. To examine differences in microbiota composition among sites and diets, beta diversity was analysed using both Weighted and Unweighted UniFrac distances, which consider both phylogenetic relationships and relative abundances of bacterial taxa. PERMANOVA (Permutational Multivariate Analysis of Variance) with 999 permutations via the beta-group-significance command in QIIME2. The --p-pairwise flag enabled pairwise comparisons across metadata groups.

Differential abundance analysis was performed using the QIIME 2 composition plugin with the ancombc method (Analysis of Compositions of Microbiomes with Bias Correction). This method applies internal FDR correction for multiple testing and identifies taxa with significant differences in abundance between groups, based on log fold-change thresholds. Comparisons were conducted based on the metadata categories *Site*, *Food*, and *SiteFood*, with reference levels specified for pairwise testing (e.g., *Honey Bee* for Food, *San Ġwann* for Site). Results were visualised using the da-barplot tool to display bacterial taxa differentially enriched across groups, with an effect size threshold of 1.5 or 2 (log fold change, LFC) depending on the comparison.

For qPCR data, normality and homogeineity of variance were assessed using Shapiro-Wilk and Levene’s test, respectively. Normally distributed data with equal variances were analysed using one-way ANOVA with Tukey HSD [41] post-hoc test. Non-normally distributed data were analysed using Kruskal-Wallis test with Dunn’s multiple comparisons post-hoc test [42–45].

For α- and β-diversity metrics and qPCR comparisons among the four sites, we applied a Bonferroni correction for 12 pairwise tests, setting the significance threshold at *p* < 0.0041. For the predicted functional data, statistical comparisons across sites with Kruskal–Wallis tests, and the resulting *p*-values were adjusted for multiple comparisons using the Benjamini–Hochberg false-discovery-rate (FDR) procedure.

## Results

### Beekeeper survey results

The beekeeper survey data collected from 2022 to 2024 provide insights into the perceived impact of *V. orientalis* on apiculture in Malta. A total of 58 beekeepers responded in 2022, 34 in 2023, and 37 in 2024. In all three years, 100% of respondents reported sightings of *V. orientalis* in their apiaries.

The proportion of beekeepers reporting colony losses due to hornet predation varied over the three-year period: 63.8% in 2022 (*n* = 37), 29.4% in 2023 (*n* = 10) and 59.5% in 2024 (*n* = 22). Reported intervention efforts, such as trapping of queens and/or drones, and manual removal, were also examined. In 2022, 82.8% of respondents (*n* = 48) reported taking action against the hornet, compared to 73.5% (*n* = 25) in 2023 and 75.7% (*n* = 28) in 2024. The effectiveness of these measures was perceived to decline over time. In 2023, 52.9% (*n* = 18) of respondents considered their interventions effective, whereas by 2024, only 43.2% (*n* = 16) did. Meanwhile, the percentage of beekeepers reporting unsuccessful control efforts rose from 11.8% (*n* = 4) in 2023 to 18.9% (*n* = 7) in 2024. A full breakdown of survey responses translated questions and annual summaries are provided in Appendix 1 - A1.1 Survey Questions and Answers.

### Gut microbiota composition and core community structure

To characterise the gut microbial communities of *V. orientalis*, we performed high-throughput sequencing of 16S rRNA gene amplicons from individual hornets collected across four sites in Malta. The following section describes the overall taxonomic composition of the hornet gut microbiota, as well as the identification of consistently prevalent core microbial taxa at both the family and genus levels.

High-throughput sequencing of *V. orientalis* gut samples generated a total of 2,964,602 clean paired-end reads, with individual samples ranging from 16,710 to 83,316 reads. After quality filtering, denoising, and merging using the DADA2 plugin, a total of 7,309 Amplicon Sequence Variants (ASVs) were identified. All samples were rarefied to a uniform sequencing depth of 16,710 merged reads for diversity analysis, and no samples were excluded at this stage.

**Fig. 1:**
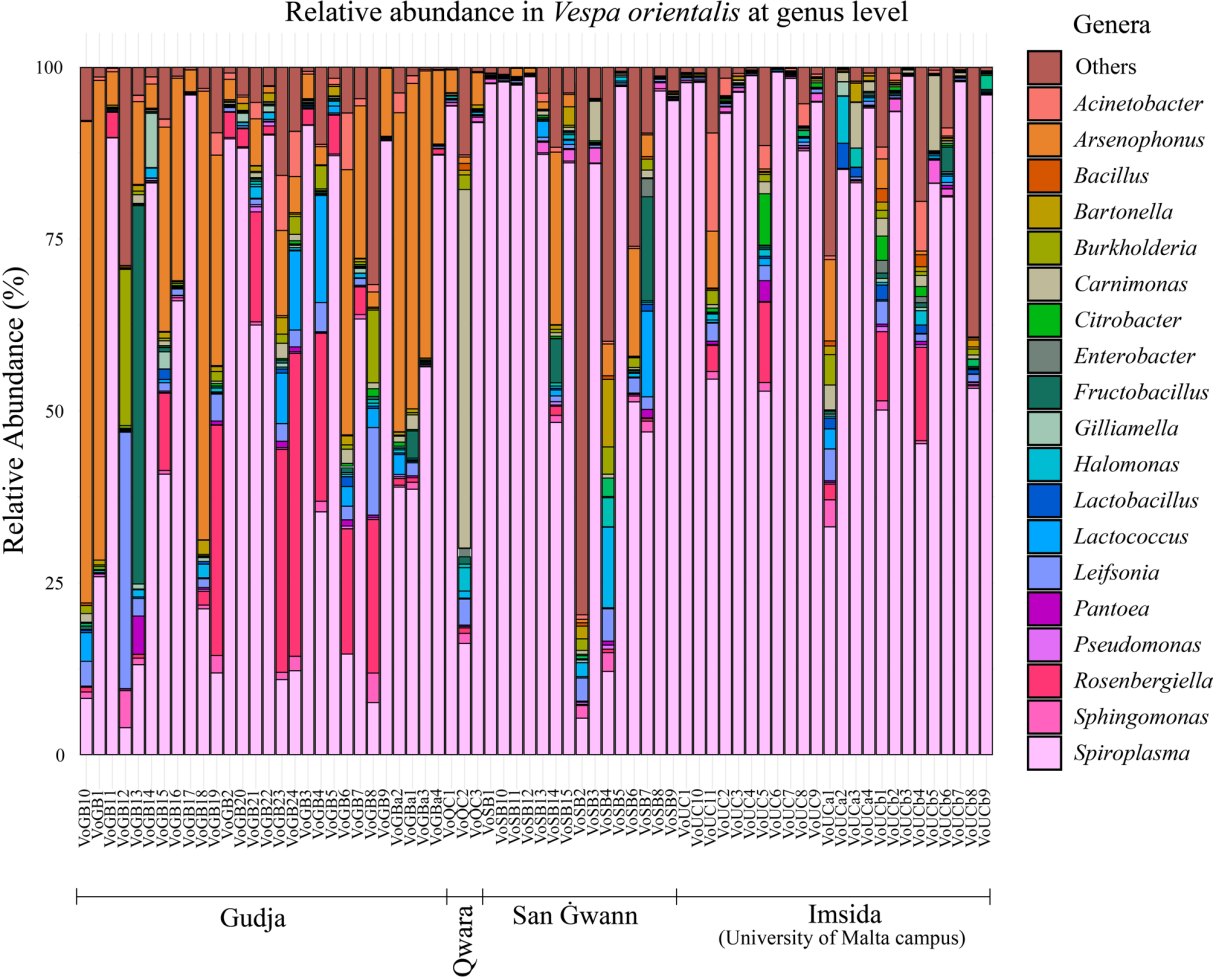
Relative abundance of the bacterial gut microbiota of *V. orientalis* at genus level. Genera that had a relative abundance of less than 1% in all samples were labelled as ‘Other’

Phylogenetic classification revealed that the gut microbiota of *V. orientalis* was predominantly composed of Mycoplasmatota (67.06% relative abundance), followed by Pseudomonadota (22.22%), Bacillota (4.75%), Actinomycetota (1.91%), Bacteroidota (0.70%), and Cyanobacteriota (0.41%). Other phyla collectively accounted for 2.95% of the total relative abundance. At the family level, the most dominant taxa were Spiroplasmataceae (67.05%), Morganellaceae (9.50%), Erwiniaceae (4.55%), and Leuconostocaceae (2.18%) (Supplementary Figure S6). At the genus level, the most abundant taxa included *Spiroplasma* (67.05%), *Arsenophonus* (9.50%), and *Rosenbergiella* (4.22%) (Fig. 1). A full summary of relative abundance percentages at the phylum, family, and genus levels is provided in Supplementary Table S4.

**Fig. 2:**
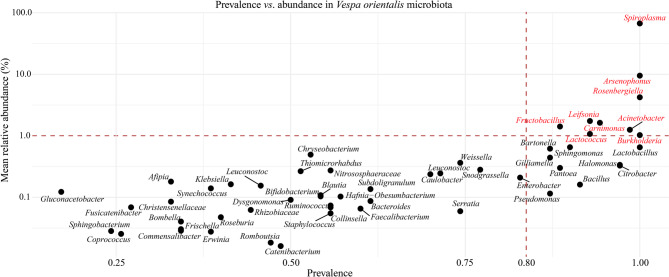
Scatter plot illustrating the prevalence and mean relative abundance of bacterial genera within the *V. orientalis* gut microbiota. Each point represents a genus, positioned according to its frequency across samples (prevalence) and its average proportion (abundance). The red dashed lines mark the chosen cutoff for both parameters, while the taxa marked in red represent the core microbiome of *V. orientalis*

To identify the most consistently present and abundant taxa, we defined the core gut microbiota at both the family and genus levels. Core taxa were defined based on prevalence score (PS) and abundance thresholds across all samples (Supplementary Table S5). At the family level, the core microbiota included Spiroplasmataceae (PS = 1.00), Morganellaceae (PS = 1.00), Halomonadaceae (PS = 1.00), Erwiniaceae (PS = 1.00), Burkholderiaceae (PS = 1.00), Moraxellaceae (PS = 0.99), Leuconostocaceae (PS = 0.99), and Microbacteriaceae (PS = 0.93) (Supplementary Figure S7). At the genus level, core taxa included *Carnimonas* (PS = 0.94), *Halomonas* (PS = 0.97), *Arsenophonus* (PS = 1.00), *Fructobacillus* (PS = 0.89), *Spiroplasma* (PS = 1.00), *Lactococcus* (PS = 0.93), *Acinetobacter* (PS = 0.99), *Rosenbergiella* (PS = 1.00), *Burkholderia* (PS = 1.00), and *Leifsonia* (PS = 0.93) (Fig. 2). A heatmap of core genera across all samples illustrates the variation in relative abundance among sites is reported in Supplementary Figure S8.

### Core microbial community predicted functionality

Hornets’ gut microbiota functionality revealed distinct profiles across individual insect samples (Supplementary Figure S9, reports the top 20 KEGG most representative metabolisms per sample). On average, amino acid synthesis accounted for 35–55% of the total functional potential, followed by protein degradation (15–25%) and monosaccharide metabolism (8–15%). In contrast, categories such as vitamin biosynthesis, alkaloid degradation, and hormone metabolism each represented less than 3%.

Moreover, aggregation of predicted functionality by sampling site reduced individual variability and revealed site-specific trends. For instance, nitrogen metabolism was particularly enriched in samples from the San Ġwann site (averaging 8.9%), while toxic compound resistance reached its highest values in Imsida samples (up to 17.4%). The predicted score value (ps) and the relative percentage are reported in Supplementary Table S6. Predicted functionality based on core gut microbiome taxa KEGG pathway coverage confirmed consistent metabolic profiles across insect samples, although with no statistically significant differences detected between sampling sites (FDR-adjusted *p* > 0.05). Nevertheless, visualisation of the top 20 pathways trends in inter-individual variability (Supplementary Figure S10).

A comparative analysis of enzymatic activities across core gut microbiome taxa revealed marked functional specialisation (Supplementary Figure S11). *Burkholderia* exhibited the highest overall enzymatic abundance, particularly for proteases (112.0), putrescine pathway enzymes (10.33), and haemolisins (4.00), suggesting a broad catabolic potential. *Arsenophonus* also showed high activity for proteases (78.33) and was the dominant producer of putrescine-pathway enzymes (8.33), with notable haemolytic capacity (3.00). In contrast, genera such as *Acinetobacter* and *Leifsonia* exhibited more limited functionality, with relatively lower enzyme counts across most categories. *Leifsonia* was an exception for hemicellulase activity (2.33).

**Fig. 3:**
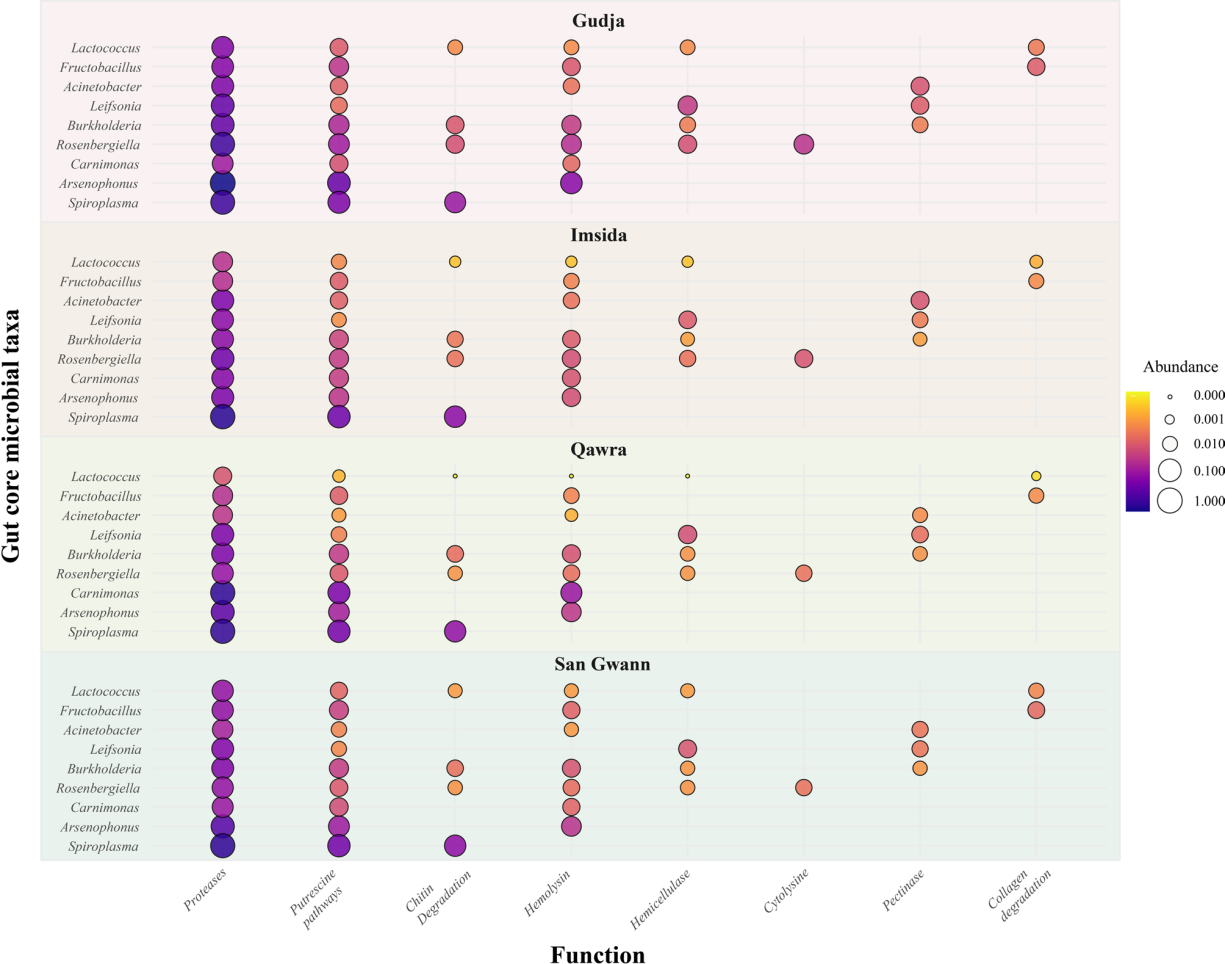
Predicted score values of core gut microbiome taxa functionality associated with putative animal tissue degradation functions (e.g., chitin degradation, collagen degradation, haemolysin, proteases, etc.) in samples collected from four Maltese locations: San Gwann, Qawra, Imsida, and Gudja. Bubble dot represent the relative abundance (log scale) of the main microbial taxa involved in different functional pathways

Considering differences across sampling sites, taxa functional contributions variations suggest local environmental filtering (Fig. 3). *Spiroplasma* exhibited consistently high relative enzyme abundance across all locations, especially in Imsida and San Ġwann, driven by putrescine pathway and protease functions. *Arsenophonus* dominated in Gudja, contributing over 1.0 relative units of proteolytic activity and exhibiting haemolytic potential. In contrast, *Carnimonas* displayed the highest protease values in Qawra, with limited activity elsewhere. While *Burkholderia* retained moderate functionality across all sites, its enzymatic activity peaked in Gudja. Notably, *Acinetobacter*, despite being widespread, showed consistently low functional profiles. However, no statistical differences were highlighted for all comparisons across sites (Mann-Whitney U test, all *p* = 1).

### Landscape impact on the gut microbial community

Alpha-diversity was assessed using Faith’s Phylogenetic Diversity (Faith PD), Observed Features and Pielou’s Evenness (Supplementary Figures S12, S13, and S14). Statistical comparisons were performed using Kruskal-Wallis H test. No significant differences in species richness or evenness across sites (Supplementary Table S7) except for Faith PD values, which differed significantly between San Ġwann and Imsida (*p* < 0.05). Beta-diversity analysis using Weighted UniFrac distances showed that gut microbial composition significantly differed by location (PERMANOVA: *p* = 0.001) (Supplementary Figure S15). Pairwise comparisons showed significant differences between Gudja vs. Imsida (*p* < 0.01) and Gudja vs. San Ġwann (*p* < 0.05) (Supplementary Table S8). Principal Coordinates Analysis (PCoA) based on Weighted UniFrac distances was used to visualise differences in gut microbial community composition among hornets from the four sampling sites (Supplementary Figure S16). The distribution of points indicates some site-level separation, with varying degrees of within-site dispersion.

Statistical comparisons of taxonomic abundances across sites were performed using Kruskal-Wallis H test with Holm-Bonferroni correction where needed. At the phylum level, Mycoplasmatota were significantly more abundant in Imsida compared to Gudja (*p* < 0.05), while Pseuodomonadota showed a higher abundance in Gudja than in San Ġwann (*p* < 0.05) and Imsida (*p* < 0.01). At the family level, Erwiniaceae and Morganellaceae were more abundant in Gudja hornets than in San Ġwann (*p* < 0.05 for both) and Imsida (*p* < 0.05 and *p* < 0.01, respectively). On the other hand, Spiroplasmataceae levels were lower in Gudja compared to Imsida (*p* < 0.05).

At the genus level, *Arsenophonus* and *Rosenbergiella* were significantly enriched in Gudja hornets compared to those in San Ġwann (both *p* < 0.05) and Imsida (*p* < 0.01 and *p* < 0.05, respectively). Conversely, *Spiroplasma* levels were lower in Gudja compared to Imsida (*p* < 0.05).

Differential Abundance Analysis (DAA) revealed site-specific differences in microbial taxa. In Gudja vs. Imsida, *Arsenophonus* was significantly more abundant in Gudja, whereas *Spiroplasma*, *Hafnia-Obesumbacterium*, *Serratia*, and *Enterobacter* were more common in Imsida samples (Log fold change = ± 3, Supplementary Figure S17). Comparisons between Gudja and San Ġwann showed that *Arsenophonus*, *Rosenbergiella*, *Dysgonomonas*, *Gluconobacter*, and *Acinetobacter* were enriched in Gudja, while *Hafnia-Obesumbacterium* was significantly higher in San Ġwann (Log fold change = ± 3, Supplementary Figure S18). Similarly, in San Ġwann vs. Imsida, *Thiomicrorhabdus*, *Blautia*, *Staphylococcus*, *Commensalibacter*, and *Proteus* were enriched in San Ġwann, while *Acinetobacter* was depleted (Log fold change = ± 3, Supplementary Figure S19). Finally, hornets sampled in the Qawra site showed an enrichment of more than 15 microbial genera, including *Carnimonas*, *Arsenophonus*, *Frischella*, *Snodgrassella*, *Gilliamella*, and *Enterobacter*, compared to hornets from San Ġwann and Gudja (Log fold change = ± 3.5, Supplementary Figure S20 and S21, respectively).

### Effect of diet on the gut microbial community

Weighted UniFrac (beta-diversity) analysis revealed a strong difference in gut microbiome composition between honey bee-associated and cat food-associated hornets (PERMANOVA, *q* = 0.006). Phylum, family and genus level differences between scavenging groups were assessed using the Kruskal-Wallis H test. At the phylum level, Mycoplasmatota were significantly more abundant in hornets consuming cat food (*p* < 0.05), while Pseudomonadota were more prevalent in those scavenging on honey bees (*p* < 0.05). At the family level, *Morganellaceae* was enriched in honey bee-associated hornets (*p* < 0.01), whereas cat food-fed hornets exhibited a higher relative abundance of *Spiroplasmataceae* (*p* < 0.05). At the genus level, this trend was reflected in the higher relative abundance of *Arsenophonus* in honey bee-associated hornets (*p* < 0.01) and *Spiroplasma* in cat food-associated hornets (*p* < 0.05).

Differential abundance analysis (DAA) further highlighted key microbial differences based on diet. *Enterobacter* was significantly more abundant in hornets consuming cat food, while *Arsenophonus* was enriched in honey bee-associated hornets (Log fold change = ± 3, Supplementary Figure S22). Additionally, significant microbiome differences were detected between hornets scavenging cat food in Qawra and those in Imsida. Specifically, *Arsenophonus*, *Gilliamella*, *Weissella*, and *Fructobacillus* were more abundant in Qawra, whereas *Planococcus*, *Salinimicrobium*, and *Snodgrassella* were more prevalent in Imsida (Log fold change = ± 3).

The relative abundance and presence of the honey bee-associated genera *Gilliamella*, *Snodgrassella*, and *Bifidobacterium* were further assessed. All three genera were detected in both honey bee-associated and cat food-associated individuals. However, no significant differences were found between the two groups in terms of relative abundance (Mann-Whitney U test, all *p* > 0.48) or presence/absence frequency (Fisher’s exact test, all *p* > 0.46). These findings suggest that the presence of these taxa is not exclusive to hornets preying on honey bees.

### Gut Microbiome total bacteria and pathogens load

**Fig. 4:**
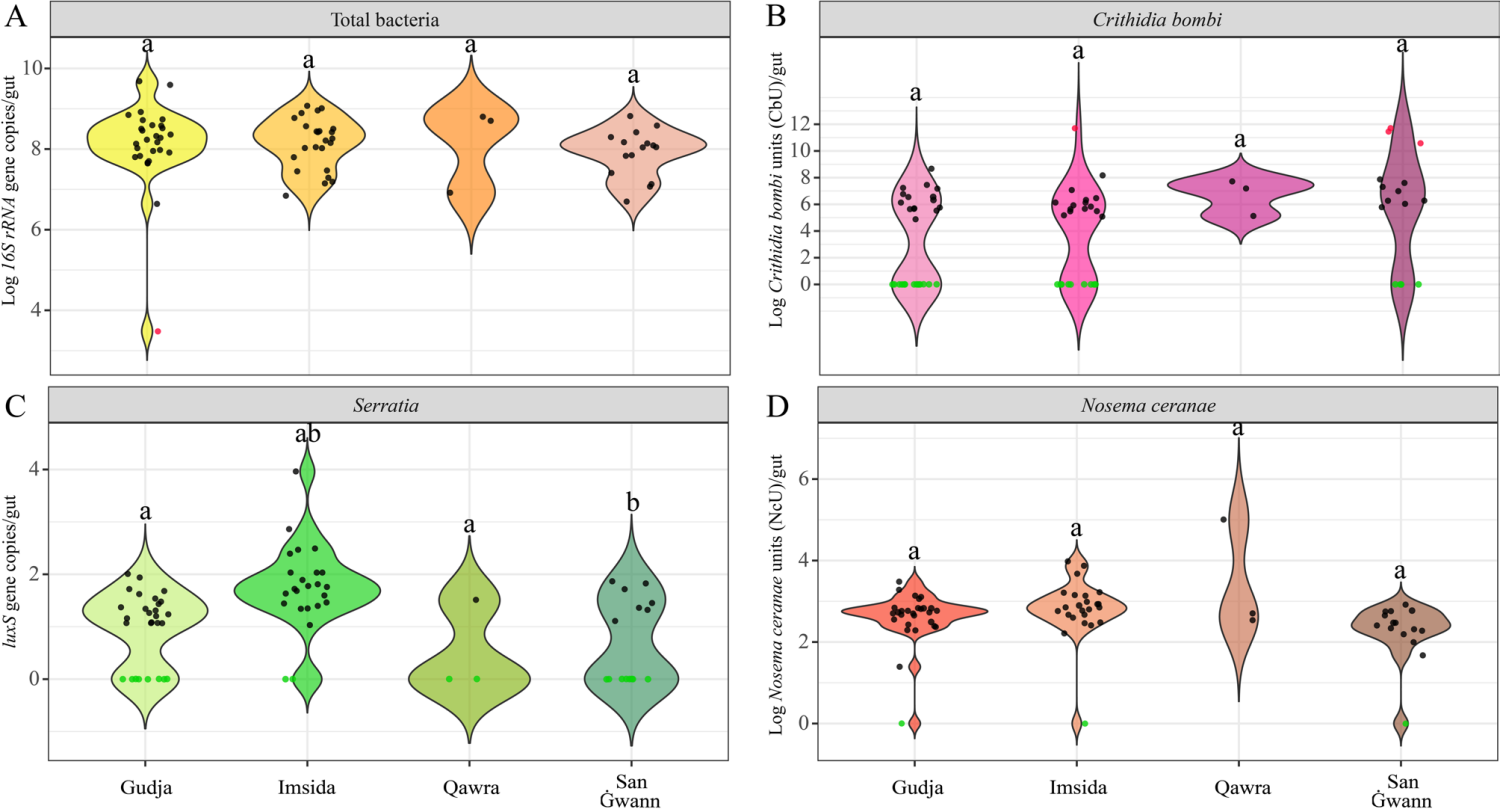
Violin plot showing qPCR results on (**A**) total bacteria; (**B**) *C. bombi*; (**C**) *Serratia*, and (**D**) *N. ceranae* detected in the *V. orientalis* gut microbiome sampled in the different Maltese sites. Different letters on the top of the violin plot indicate a *p* < 0.05. Red circles indicate outliers, whereas green circles indicate negative samples for the target pathogen

Sample sizes for qPCR analyses matched those used in sequencing, with site-level disparities: Gudja (*n* = 28), Imsida (*n* = 24), San Ġwann (*n* = 15), and Qawra (*n* = 3). qPCR results showed that total bacterial load in the hornet gut microbiota ranged between Log 6 and Log 9 copies per individual (Fig. 4A), with no significant differences observed across sampling sites. *N*. *ceranae* was detected in 97.15% of the sampled hornets with an average absolute abundance of Log 2.64 ± 0.75 spores per gut (Fig. 4B). *Serratia* (targeting the *luxS* gene) was found in 71.43% of individuals and showed significantly higher abundance in hornets from Imsida (1.76 ± 0.81 *luxS* gene copies/gut) compared to Gudja (0.99 ± 0.68 *luxS* gene copies/gut) and San Ġwann (0.71 ± 0.81 *luxS* gene copies/gut) (*p* < 0.05, Fig. 4C). *C bombi* was detected in 62.86% of individuals and exhibited the widest range in abundance, with a mean of Log 4.29 ± 3.58 (Fig. 4D). The load of *C*. *bombi* and *N*. *ceranae* did not differ significantly among the four sites. Both pathogens were detected in hornets from the urban locations of Imsida and Qawra, where the insects were scavenging cat food rather than engaging in honey bee predation. This shows that hornets can carry these pathogens even when they have not been observed attacking honey bee colonies. None of the other screened pathogens *N*. *apis*, *N*. *bombi*, *C*. *mellificae*, *L*. *passim*, *A*. *bombi*, *Listeria*, or *Salmonella*, were detected in any sample. All the qPCR results are reported in Supplementary Table S9.

## Discussion

While the gut microbiota of hornets has begun to receive attention, few studies have linked microbial variation to ecological context, such as foraging behaviour, seasonality, and landscape use. In this study, *V*. *orientalis* individuals were sampled during the active foraging season (September - October), the period when hornet predation on honey bees is most intense in Malta. Hornets were collected from four sites representing two distinct foraging and land-use contexts: (i) active honey bee predation at two apiary locations (Gudja and San Ġwann) and (ii) protein scavenging on leftover cat food (used here as a proxy for anthropogenic protein sources) at two urban/peri‑urban locations (Qawra and Imsida). These conditions provided a natural framework to explore how diet and environment shape gut microbial composition. To contextualise microbiome findings and better understand the ecological impact of *V. orientalis*, we also incorporated a three-year beekeeper survey. Respondents consistently reported hornet sightings and seasonal honey bee colony losses, particularly during the summer and early autumn months, the same window covered by our sampling. These field observations validated our site-selection strategy, confirming that the chosen apiary sites experience high hornet pressure and, therefore, an increased likelihood of hornet-mediated microbial exchange. They also illustrate why this native hornet is now perceived as behaving invasively in Malta. The functional predictions and pathogen screening presented in this study remain exploratory and hypothesis-generating, aimed at identifying microbial patterns and putative transmission pathways that merit more focused investigation in future work. Full details and summary data from the survey are provided in Appendix 1 - A1.2 Survey Interpretation and Discussion.

### Composition of the gut microbiota in *V. orientalis*

The gut microbiota of *V. orientalis* was dominated by Mycoplasmatota and Pseudomonadota, consistent with findings in previous studies on other *Vespa* species [19–22]. Additionally, Bacteroidetes and Actinobacteria were present, aligning with most prior studies, except for Cini et al. [22], who did not report these phyla. However, at lower taxonomic levels, significant variation was observed, reinforcing previous reports that gut composition diverges among *Vespa* species [19]. Our results designated the main *V. orientalis* core microbiome family as Spiroplasmataceae, Morganellaceae, Halomonadaceae, Erwiniaceae, Burkholderiaceae, Moraxellaceae, Leuconostocaceae, and Microbacteriaceae. Core genera are *Carnimonas*, *Arsenophonus*, *Fructobacillus*, *Spiroplasma*, *Lactococcus*, *Acinetobacter*, *Rosenbergiella*, *Burkholderia*, and *Leifsonia*. Of these genera, the most abundant in *V. orientalis* were *Spiroplasma* and *Arsenophonus.* Interestingly, *Spiroplasma* has been reported in *Vespa* only in a recent study by Hettiarachchi et al. [20], while *Arsenophonus* has not been previously documented in hornet gut microbiota [19]. Several genera detected in our samples, *Fructobacillus*, *Leuconostoc*, *Lactococcus*, *Weissella*, *Gilliamella*, *Carnimonas*, *Snodgrassella*, and *Pantoea*, have likewise been reported in earlier *Vespa* gut microbiome studies [19], even though not all of these genera surpassed our core microbiota threshold.

### Drivers of gut microbiota variation: diet and geography

Phylogenetic diversity metrics (Faith’s PD, Observed Features, Pielou’s evenness) revealed no significant differences in alpha diversity between honey bee-associated and cat food-associated hornets, suggesting that overall microbial richness and evenness were relatively stable across dietary groups. This is in contrast with Suenami et al. [19], who proposed that hornet core microbiota is primarily shaped by diet. While our alpha diversity results do not support this, compositional differences at the genus level suggest that some specific taxa are influenced by dietary input.

Notably, honey bee-associated hornets exhibited a marked enrichment in *Arsenophonus*. A known honey bee endosymbiont [46], *Arsenophonus*, is a genus that has previously been found in relatively high abundance in Maltese honey bees [25]. *Arsenophonus* has been reported at elevated prevalence in colonies suffering from Colony Collapse Disorder (CCD) in the United States [47] and was singled out by Budge et al. [48] as one of the strongest bacterial predictors of poor colony performance in a nationwide survey of British apiaries. *Arsenophonus* has also been detected in insect-eating arthropods such as the wolf spider *Pardosa pseudoannulata* and the ladybird *Novius pumilus*, where horizontal transmission via prey is suspected [49, 50]. Finding this genus in *V*. *orientalis* therefore may reflect acquisition through predation on infected bees or through shared floral resources [25, 48–50]. Because 16S rRNA amplicon sequencing could not resolve species‑level identity within this genus, the precise origin and function of *Arsenophonus* in *V. orientalis* remain uncertain. Its presence hints at microbial transfer from prey, but this interpretation should be viewed cautiously.

In contrast, hornets scavenging on cat food exhibited a higher relative abundance of *Enterobacter*, a genus commonly associated with decomposing protein sources such as raw meat and spoiled pet food [51, 52]. *Enterobacter* has previously been isolated from the gut of *Vespa velutina nigrithorax* [53] and has also been found in other insects, including tsetse and fruit flies [54, 55]. Its prevalence in scavenging hornets supports the influence of diet and resource type on gut microbial composition.

Other bacteria generally associated with honey bees, such as *Gilliamella*, *Snodgrassella*, and *Bifidobacterium*, were detected in hornets from both dietary groups. Statistical analysis revealed no significant differences in their relative abundance or presence/absence between bee- and cat food-associated individuals (all *p* > 0.48). Their ubiquity suggests multiple acquisition routes, past predation, environmental exposure, or overlapping foraging, which may explain the substantial shared microbiome despite distinct primary food sources. Taken together, our results indicate that diet does not markedly alter the overall evenness or richness of the *V*. *orientalis* gut microbiota, likely because opportunistic foraging produces a broadly shared community across individuals. Yet two taxa stand out: *Enterobacter* is enriched in hornets scavenging on anthropogenic meat sources, whereas *Arsenophonus* is enriched in hornets preying on honey bees. Beyond diet, community-level analyses revealed significant geographic differences in microbial composition. Weighted UniFrac distances indicated that hornets from Gudja (a honey bee-associated site) harboured distinct gut microbiota compared to those from both Imsida (a cat food-associated site) and San Ġwann (another honey bee-associated site). The divergence between Gudja and San Ġwann, despite similar observed predatory behaviour, suggests that landscape features, floral diversity, or environmental microbial exposure may shape gut communities independently of diet [56, 57]. Future comparative studies incorporating sympatric, non-predatory insects that share foraging habitats with *V. orientalis*, but do not prey on honey bees, would help disentangle the effects of shared environment from those of trophic interactions.

### Pathogen detection and ecological implications

Our qPCR screening detected three bee-associated microbial taxa in *V*. *orientalis*: *N*. *ceranae* (97.15% prevalence), *C*. *bombi* (62.86%), and *Serratia* (71.43%). The remaining bee-associated targets: *N. apis*, *N. bombi*, *C. mellificae*, *L*. *passim*, and *A*. *bombi* were not detected. While *Serratia*, previously isolated from *V. orientalis* by Zucca et al. [16], occurred at low abundance, it was significantly more prevalent in individuals from Imsida, an urban site. While some *Serratia* strains are opportunistic pathogens in humans, this genus is common in soil, water, and insects [58, 59], and its detection here more likely reflects environmental exposure than any direct relevance to human health. *Listeria* and *Salmonella* were not detected in any samples, consistent with prior findings in *V. orientalis* [16], suggesting the species is unlikely to be a meaningful carrier of these pathogens under current conditions.

The detection of the two well-established bee-pathogens, *N. ceranae* and *C. bombi*, in a substantial proportion of hornets from both studied associated-diets, suggests that acquisition is not limited to active predation. Although Zucca et al. [16] did not detect *N*. *ceranae* in the *V*. *orientalis* individuals they examined, the parasite has been reported in this hornet elsewhere [60] and in both *V. velutina nigrithorax* and *V. crabro* [61] [16, 61]. Moreover, *C*. *bombi* was also previously detected in *V. orientalis* [60] and in *V. velutina nigrithorax* and *V. crabro* [61]. The presence of these pathogens in non-bee-feeding individuals suggests additional acquisition pathways, such as contact with contaminated floral resources or other insect prey. This remains a hypothesis; targeted environmental DNA or pollen wash screening of the plants frequented by both hornets and bees will be needed to confirm the role of shared flowers as a transmission hub [62–64]. These results broaden our view of *V. orientalis* in pathogen dynamics. While hornets have previously been considered vectors capable of transmitting pathogens to honey bees (spillover) [23, 64, 65], our detection of bee‑associated microbes, even in cat food‑scavenging individuals, implies additional processes. One possibility is environmental spillback, where pathogens originating from bees persist on shared substrates (flowers, soil, carcasses), through which they are picked up by the hornets and are later re-transmitted to bees via hornet contact [62, 63]. Alternatively, hornets may act as mechanical carriers, transporting pathogens acquired from prey without internal replication. Although our 16S data cannot confirm viability, the presence of these taxa across diet groups highlights the need to test whether hornet‑mediated redistribution contributes to apiary‑scale disease pressure.

Building on this, the sporadic detection of bee‑associated microbes across our limited sample set indicates that hornets may acquire these taxa opportunistically and carry them passively rather than sustaining persistent infections. We did not capture a full spillback cycle, but the hypothesis remains plausible. Crucial next steps are to determine (i) whether the microbes remain viable after gut passage, and (ii) whether hornets shed them via faeces, regurgitated droplets, or contaminated surfaces shared with bees. Other insect predators can excrete viable pathogens after ingestion [23, 65, 66] assessing whether *V. orientalis* does should be a priority for future work [23, 65, 66]. Floral contact offers a plausible acquisition route. *Nosema ceranae* spores can persist on flowers and move between *Apis mellifera* and *Tetragonula hockingsi* [67], while infective levels of *C*. *bombi* have been detected on blossoms after deposition by bees and flies [68]. Such findings raise the possibility that *V. orientalis* picks up pathogens indirectly from shared floral resources, not only through predation. Our detection of *C. bombi*, a parasite typically associated with bumble bees, also hints at interactions with a broader pollinator guild. Although predation on honey bees is well documented, other *Vespa* species (e.g., *V. velutina nigrithorax* preying on *B*. *terrestris* [69]) show that hornets can exploit bumble bees as well, suggesting similar behaviour may occur in *V. orientalis*.

Determining the hornet’s functional role now requires targeted work on pathogen viability, replication, and shedding. Controlled feeding experiments that track microbial survival post‑ingestion, coupled with assays for excretion or surface contamination, would clarify whether hornets actively recycle pathogens or simply mirror incidental exposure. Such data are increasingly important as *V. orientalis* expands its range and overlaps more heavily with both managed and wild pollinators [67–69].

### Ecological and predicted functional insights into the gut Microbiome of *V. orientalis*

This study presents the first in-depth predicted functional profile of the *V*. *orientalis* gut microbiome, revealing a structured and metabolically versatile community inferred from the genomes of its core bacterial taxa. Functional predictions based on enzyme commission (EC) number mapping indicate that the microbiota is dominated by pathways involved in amino acid synthesis, protein degradation, and monosaccharide metabolism. Bubble‑plot analysis of the top 20 KEGG pathways (Supplementary Figure S9) revealed a metabolically robust and largely uniform gut community. Core functions, amino acid biosynthesis, vitamin/co‑factor production, and central carbon metabolism, were highly represented in every individual, regardless of diet. Amino acid synthesis accounted for over half (55%) of predicted activity. Although *V*. *orientalis* inhabits a protein-rich ecological niche, it is important to consider the dietary behaviour of adult hornet (female) workers. Despite engaging in predation or scavenging, adult *V. orientalis* workers primarily ingest carbohydrate-rich fluids such as nectar and larval secretions, with limited direct protein consumption. These larval secretions, while rich in carbohydrates and some free amino acids [70, 71], may not consistently provide a complete profile of essential amino acids. Therefore, the prominence of microbial amino acid synthesis pathways likely reflects a compensatory microbial function that enhances nutritional resilience in the face of fluctuating or incomplete amino acid availability. In this context, microbial synthesis of amino acids can support both microbial maintenance and host metabolism, particularly for tissue development or physiological demands such as foraging and venom production. These predicted pathways may also act as a buffer when environmental food sources are limited or variable, as might occur in urban settings or late in the foraging season.

To further explore the trophic ecology of *V. orientalis* workers, we centred our analysis on eight enzyme-based functional categories that are mechanistically linked to the prey- and nectar-derived nutrients these hornets routinely exploit. Focusing on proteolysis, chitin, polyamine and cytolytic modules therefore captures the functions the gut community is most likely to express. Across the gut community of the hornet, protease was the most abundant metabolic function (with the most abundant being in Gudja, an apiary site). Three genera dominated this activity; *Burkholderia*, *Arsenophonus* and *Rosenbergiella*, and are detected at every site, indicating a core enzymatic backbone that transcends geography and dietary source. Taken together, these enzyme classes greatly outnumber plant-carbohydrate and toxin functions, highlighting that the hornet gut microbiome is geared primarily toward efficient extraction of protein from prey or other protein food sources. Although adult hornet workers primarily consume nectar and larval secretions as sources of carbohydrates and free amino acids, evidence suggests they also possess the enzymatic capacity to metabolise dietary proteins [69, 72, 73]. These findings support the view that while worker adults do not collect dietary protein for self-consumption, they do ingest and break down incidental protein during protein processing.

Following protease functional category, chitin degradation and putrescine pathways make up the next most abundant tier of predicted functions. Chitin is a minor share, while putrescine pathways takes up a larger share of the metabolic capacity. Chitin scores are highest in the hornets collected from Imsida (urban), followed by San Ġwann (apiary) and Qawra (urban), with Gudja (apiary) last. Putrescine potential is comparable across sites, with *Arsenophonus*, *Carnimonas* and *Spiroplasma* dominating.

Collagen‑degradation genes represent only a trace component of the functional pool and appear almost exclusively in rare reads attributed to *Fructobacillus* and *Lactococcus*, with sporadic presence, even in the hornets that have regular access to cat food waste. Pectin‑ and hemicellulose‑hydrolysing enzymes are likewise scarce across all hornets. Although their abundance varies modestly from site to site, no consistent difference emerges between urban and apiary settings. The taxa that contribute most, *Acinetobacter* and *Leifsonia*, appear to provide a low‑level capacity to tap plant‑derived sugars. Haemolysin genes peak in Gudja thanks to *Arsenophonus*, while Qawra shows a smaller spike, driven by *Carnimonas*. Cytolysin genes follow the same pattern.

Taken together, the data portray a gut microbiome whose protein‑scavenging core seems stable, emphasising that prey‑derived chitin and nitrogen recycling remain central no matter where hornets feed. No statistically significant differences in pathway-level predictions were detected, suggesting a stable core metabolic framework, likely reflecting mixed prey and waste-derived diets.

## Conclusion

This study presents the first comprehensive characterization of the gut microbiota of *V. orientalis*, integrating taxonomic, functional, and pathogen screening data to examine the ecological dynamics of this expanding predator. While dietary sources and environmental context influenced microbial composition, evidenced by *Arsenophonus* enrichment in honey bee-associated hornets and *Enterobacter* in scavenging individuals, a stable core microbiota and conserved functional potential were maintained across individuals and sites. Predicted functions were dominated by amino acid synthesis and protein digestion, supporting a metabolically versatile gut community well-adapted to the hornet’s varied foraging behaviour. The detection of bee-associated pathogens (*N ceranae* and *C. bombi*) in both predatory and scavenging hornets supports the hypothesis that *V. orientalis* may acquire pollinator-associated microbes through environmental contact, floral contamination, or prey ingestion. These acquisition routes were not experimentally tested in this study, which was observational and based on 16S rRNA gene profiling. Confirming such pathways would require targeted experiments, such as pathogen viability assays, floral exposure tests, or gut acquisition studies, to assess whether these microbes remain viable and transmissible. Our data also does not confirm whether *V. orientalis* acts as a functional or transient carrier, and further research is needed to assess microbial viability, replication, and transmission potential. Moreover, given the limited number of individuals sampled at certain sites and the inherent ecological differences between them, our conclusions regarding the influence of diet and site on microbial composition should be interpreted with caution. Controlled studies, such as experimental feeding trials or comparisons between sexes (e.g., males vs. foraging females), would be needed to disentangle dietary versus environmental effects more robustly. Taken together, these results remark the ecological adaptability of *V. orientalis* and its potential to intersect with pollinators through shared microbial exposures.

## Supplementary Information

Below is the link to the electronic supplementary material.

Supplementary Material 1

Supplementary Material 2

## Data Availability

NGS raw sequence data have been submitted to NCBI repository under the Sequence Read Archive (SRA) databases under the Bioproject N° PRJNA1232968, biosamples SAMN47255446 - SAMN47255585. Moreover, multimedia material on *V. orientalis* are available on Mendelay data repository at the following DOI: 10.17632/4ng7kx3nff.1.
