# Supplementary material for: Gut microbiome of Vespa orientalis: functional insights and potential honey bee pathogen dynamics
Source: Anim Microbiome. 2025 Sep 30;7:95. doi: 10.1186/s42523-025-00460-6 (PMC12482325; doi:10.1186/s42523-025-00460-6)
Supplement: Supplementary file 2 — Supplementary Material 2 [file 42523_2025_460_MOESM2_ESM.pdf]

Gut microbiome of *Vespa orientalis*: functional insights and potential honey bee pathogen dynamics

Simone Cutajar[a,b], Chiara Braglia[a], Daniele Alberoni[a*], Martina Mifsud[b], Loredana Baffoni[a], Jorge Spiteri[c], Diana Di Gioia[a], David Mifsud[b].

Affiliations
[a] Dipartimento di Scienze e Tecnologie Agro-Alimentari (DISTAL), University of Bologna, Viale Fanin 42, 40127, Bologna, Italy;

[b] Institute of Earth Systems, L-Università tà Malta, Msida, Malta;

[c] Malta Beekeepers' Association VO1527 c/o, Volunteer Centre, 181, Triq Melita, Valletta, Malta, VLT 1129.

Orchid ID
Simone Cutajar: https://orcid.org/0009-0002-7717-0932
Daniele Alberoni: https://orcid.org/0000-0002-2394-2880
Diana Di Gioia: https://orcid.org/0000-0002-0181-1572
Chiara Braglia: https://orcid.org/0000-0002-1637-896X
Loredana Baffoni: https://orcid.org/0000-0001-5313-5871
David Mifsud: https://orcid.org/0000-0001-9562-1077
Jorge Spiteri: NA

[*] **Corresponding Author:**
Daniele Alberoni (daniele.alberoni@unibo.it)

Keywords: *Vespa orientalis*, pollinator pathogens, pathogen spillback, functional prediction, *Arsenophonus*, *Nosema ceranae*, *Crithidia bombi, Spiroplasma*, *Enterobacter*

Table S1. Genomes used for functional study retrieved from NCBI database.

Organism	RAST genome ID	NCBI GenBank	NCBI RefSeq
Acinetobacter baumannii ATCC 19606	470.29639	GCA_020268605.1	GCF_020268605.1
Acinetobacter pollinis SCC477	2605270.1	GCA_015627175.1	GCF_015627175.1
Acinetobacter baretiae B10A	2605383.4	GCA_015627105.1	GCF_015627105.1
Acinetobacter nectaris CIP110549	1219382.9	GCA_000488215.1	GCF_000488215.1
Arsenophonus apicola ArsBeeUs	6666666.103	GCA_020268605.1	GCF_020268605.1
Arsenophonus apicola aApi_AU	2879119.1	GCA_029906405.1	GCF_029906405.1
Arsenophonus nasoniae FIN	638.36	GCA_004768525.1	GCF_004768525.1
Burkholderia gladioli BBB-01	28095.426	GCA_016698705.1	GCF_016698705.1
Burkholderia cepacia ATCC 25416	292.731	GCA_006094315.1	GCF_006094315.1
Burkholderia pseudomultivorans LMG 26883	1207504.38	GCA_902499075.1	GCF_902499075.1
Carnimonas nigrificans ATCC BAA-78	64323.3	GCA_000526695.1	GCF_000526695.1
Fructobacillus evanidus LMG_32999	3064281.1	GCA_963580165.1	GCF_963580165.1
Fructobacillus apis W13	2935017.9	GCA_023893635.1	GCF_023893635.1
Fructobacillus fructosus KCTC 3544	1631.3	GCA_000185045.2	GCF_000185045.1
Fructobacillus fructosus NRIC_1058	1631.29	GCA_001047095.2	GCF_001047095.2
Lactococcus garvieae ASM1602669v1	1363.55	GCA_016026695.1	GCF_016026695.1
Lactococcus insecticola Hs20B0	2709158.7	GCA_011170065.1	GCF_011170065.1
Lactococcus nasutitermitis NRBC111537	1652957.12	GCA_018403765.1	GCF_018403765.1
Lactococcus termiticola NtB2	2169526.1	GCA_003116835.1	GCF_003116835.1
Lactococcus lactis DSM 20481	1360.889	GCA_029023865.1	GCF_029023865.1
Lactococcus plantarum NBRC 100936	1365.23	GCA_001591745.1	GCF_001591745.1
Leifsonia aquatica DSM 20146	144185.12	GCA_014190775.1	GCF_014190775.1
Leifsonia poae VKM Ac-1401	110933.1	GCA_027921845.1	GCF_027921845.1
Leifsonia flava SYP-B2174	2561933.13	GCA_004570845.1	GCF_004570845.1
Rosenbergiella australiborealis CdVSA20.1	1544696.15	GCA_018494035.1	GCF_018494035.1
Rosenbergiella collisarenosi 8.8A	1544695.18	GCA_018494085.1	GCF_018494085.1
Rosenbergiella epipactidis subsp. c*aliforniensis* FR72	3034749.3	GCA_022602615.1	GCF_022602615.1
Rosenbergiella epipactidis subsp. j*aponicus* K24	3034750.3	GCA_022602435.1	GCF_022602435.1
Rosenbergiella epipactidis 2.1A	1544694.32	GCA_018494055.1	GCF_018494055.1
Rosenbergiella gaditana S61	2726987.5	GCA_018494065.1	GCF_018494065.1
Rosenbergiella metrosideri JB07	2921185.7	GCA_022602565.1	GCF_022602565.1
Rosenbergiella nectarea FR67	988801.4	GCA_022602735.1	GCF_022602735.1
Spiroplasma monobiae MQ-1	2136.4	GCA_002865545.1	GCF_002865545.1
Spiroplasma apis B31	2137.4	GCA_000500935.1	GCF_000500935.1
Spiroplasma litorale TN-1	216942.23	GCA_001267155.1	GCF_001267155.1

Table S2. Complete list of the functional gene categories detected among the core gut microbial taxa genome annotated in RAST.

Functional Gene Category	N° of pathway
Amino Acid Metabolism	2306
Aromatic Compound Metabolism	105
Carbohydrate Metabolism	773
Defense / Resistance	753
Fatty Acid Metabolism	196
Hydrolases	4966
Hypothetical Protein	33415
Isomerases	1569
Ligases	2039
Lyases	2268
Nitrogen Metabolism	459
Other	40356
Oxidoreductases	4779
Regulation	4762
Sulfur Metabolism	747
Transferases	7643
Transport	7577
Vitamin Metabolism	26

Table S3. qPCR-specific primers used in this study. **Annealing temperature (T°) and melting temperature (T°) of undetected targets are reported based on reference information.* the letters that substitute the classic nucleotides A,T, C and G, are described here: Y= C or T; W= A or T; H= A or C or T; R= A or G.

Target	Primer Name	Sequence (5′-3′)	Amplicon size (bp)	Annealing Temperature °C	Melting °C (T_m)	Reference
Eubacteria	Eub338-Fw	ACTCCTACGGGAGGCAGCAG	200	60	-	Lane, 1991
	Eub518-Rev	ATTACCGCGGCTGCTGG				
Nosema ceranae	Nc841f	GAGAGAACGGTTTTTTGTTTGAGA	140	60	77.1	Huang and Solter, 2013
	Nc980r	ATCCTTTCCTTCCTACACTGATTG				
Nosema apis	321 apis fw	GGGGGCATGTCTTTGACGTACTATGTA	321	60*	84.3 *	Martin-Hernandez et al., 2007
	321 apis rv	GGGGGGCGTTTAAAATGTGAAACAACTATG				
Serratia	luxS1-Fw	TGCCTGGAAAGCGGCGATGG	<300	61	86.4	Joyner et al., 2014
	LuxS2-Rv	CGCCAGCTCGTCGTTGTGGT				
Nosema bombi	NBOMBI 323 fw	CCATGCATGTTTTTGAAGATTATTAT	323	56*	83.5 *	Klee and Paxton, 2015
	NBOMBI 323 rv	CATATATTTTTAAAATATGAAACAATAA				
Crithidia bombi	C.bombi_119Fw	CCAACGGTGAGCCGCATTCAGT	164	59	82	Huang et al., 2015
	C.bombi_119Rv	CGCGTGTCGCCCAGAACATTGA				
Crithidia mellificae	Cmel_Cyt_b_F	TAAATTCACTACCTCAAATTCAATAACATA ATCAT	131	59*	-	Xu et al., 2018
	Cmel_Cyt_b_	ATTTATTGTTGTAATCGGTTTTATTGGATAT GT				
Lotmaria passim	LpRPB_F	CCCATACCAGCGATCCTCA	336	59*	-	Bartolomé et al., 2018
	LpRPB_R	ATGAACTTCGCCACCTCATCA				
Apicystis bombi	ApBF1 293 fw	CGTACTGCCCTGAATACTCCAG	293	58*	81.7 *	Meeus et al., 2018
	ApBR1 293 rv	TGAAAGCGGCGTATACATGA				

Listeria	*iap*-31-deg-F	CAYCCGCWAGCACWGTAGTAGT	78	60*	75.5 –77*	Barbau-Piednoir et al., 2013
	iap-50-deg-R	GCGTCRACAGTWGTSCCHTT				
Salmonella	Styinva-JHO-Fw	TCGTCATTCCATTACCTACC	119	55*	-	Nam et al., 2005; Hoorfar et al., 2000
	Styinva-JHO-Rv	AAACGTTGAAAAACTGAGGA				

Huang, W. F., & Solter, L. F. (2013). Comparative development and tissue tropism of *Nosema apis* and *Nosema ceranae*. Journal of invertebrate pathology, 113(1), 35-41. https://doi.org/10.1016/j.jip.2013.01.001

Martín-Hernández, R., Meana, A., Prieto, L., Salvador, A. M., Garrido-Bailón, E., & Higes, M. (2007). Outcome of colonization of *Apis mellifera* by *Nosema ceranae*. Applied and environmental microbiology, 73(20), 6331-6338. https://doi.org/10.1128/AEM.00270-07

Joyner, J., Wanless, D., Sinigalliano, C. D., Lipp, E. K. (2014). Use of quantitative real-time PCR for direct detection of Serratia marcescens in marine and other aquatic environments. A.E.M. 80, 1679–1683. https://doi.org/10.1128/AEM.02755-13

Klee J, Tay WT, Paxton RJ. Specific and sensitive detection of *Nosema bombi* (Microsporidia: Nosematidae) in bumble bees (Bombus spp.; Hymenoptera: Apidae) by PCR of partial rRNA gene sequences. J. Invertebr. Pathol. 2006;91(2):98-104. https://doi.org/10.1016/j.jip.2005.10.012

Huang, W. F., Skyrm, K., Ruiter, R., & Solter, L. (2015). Disease management in commercial bumble bee mass rearing, using production methods, multiplex PCR detection techniques, and regulatory assessment. Journal of Apicultural Research, 54(5), 516-524. https://doi.org/10.1080/00218839.2016.1173352

Xu, G., Palmer-Young, E., Skyrm, K., Daly, T., Sylvia, M., Averill, A., & Rich, S. (2018). Triplex real-time PCR for detection of *Crithidia mellificae* and *Lotmaria passim* in honey bees. Parasitology research, 117, 623-628. https://doi.org/10.1007/s00436-017-5733-2

Power, K., Cilia, G., Ragusa, E., Rizzo, R., Bortolotti, L., & Maiolino, P. (2024). Occurrence of *Nosema ceranae*, *Ascosphaera apis* and trypanosomatids in *Vespa orientalis* linneus 1771. Journal of Invertebrate Pathology, 206, 108168. https://doi.org/10.1016/j.jip.2024.108168

Bartolomé, C., Buendía, M., Benito, M., De la Rúa, P., Ornosa, C., Martín-Hernández, R., ... & Maside, X. (2018). A new multiplex PCR protocol to detect mixed trypanosomatid infections in species of *Apis* and *Bombus*. Journal of invertebrate pathology, 154, 37-41. https://doi.org/10.1016/j.jip.2018.03.015

Meeus I, Pisman M, Smagghe G, Piot N. Interaction effects of different drivers of wild bee decline and their influence on host–pathogen dynamics. Curr Opin Insect Sci. 2018;26:136–41 https://doi.org/10.1016/j.cois.2018.02.007

Barbau-Piednoir, E., Botteldoorn, N., Yde, M., Mahillon, J., & Roosens, N. H. (2013). Development and validation of qualitative SYBR® Green real-time PCR for detection and discrimination of *Listeria* spp. and *Listeria monocytogenes*. Applied Microbiology and Biotechnology, 97, 4021-4037 https://doi.org/10.1007/s00253-012-4477-2

Nam, H. M., Srinivasan, V., Gillespie, B. E., Murinda, S. E., & Oliver, S. P. (2005). Application of SYBR green real-time PCR assay for specific detection of *Salmonella* spp. in dairy farm environmental samples. International journal of food microbiology, 102(2), 161-171. https://doi.org/10.1016/j.ijfoodmicro.2004.12.020

Hoorfar, J., Ahrens, P., & Rådstrom, P. (2000). Automated 5′ nuclease PCR assay for identification of *Salmonella* enterica. Journal of clinical microbiology, 38(9), 3429-3435. https://doi.org/10.1128/jcm.38.9.3429-3435.2000

Table S4. The average relative abundance of Amplicon Sequence Variants (ASVs) at the phylum, family, and genus levels.

**Phylum**		**Family**		**Genus**	
**Mycoplasmatota**	67.06%	**Spiroplasmataceae**	67.05%	***Carnimonas***	1.63%
**Pseudomonadota**	22.22%	**Morganellaceae**	9.50%	***Arsenophonus***	9.50%
**Bacillota**	4.75%	**Erwiniaceae**	4.55%	***Fructobacillus***	1.41%
**Actinomycetota**	1.91%	**Leuconostocaceae**	2.18%	***Spiroplasma***	67.05%
**Bacteroidota**	0.70%	**Halomonadaceae**	1.96%	***Lactococcus***	1.07%
**Cyanobacteriota**	0.41%	**Microbacteriaceae**	1.74%	***Acinetobacter***	1.25%
**Others**	2.95%	**Moraxellaceae**	1.25%	***Rosenbergiella***	4.22%
		**Streptococcaceae**	1.07%	***Burkholderia***	1.03%
		**Burkholderiaceae**	1.03%	***Leifsonia***	1.74%
		**Others**	9.68%	**Others genus**	11.11%

Table S5. Prevalence scores and relative abundance percentages of gut microbiome members at the family and genus levels.

Family	Prevalence	Abundance (%)	Genera	Prevalence	Abundance (%)
Acetobacteraceae	0.557	0.191	*Acinetobacter*	0.986	1.251
Bacillaceae	0.914	0.161	*Afipia*	0.329	0.180
Bacteroidaceae	0.614	0.087	*Arsenophonus*	1.000	9.498
Bifidobacteriaceae	0.543	0.103	*Bacillus*	0.914	0.161
Burkholderiaceae	1.000	1.026	*Bacteroides*	0.614	0.087
Caulobacteraceae	0.700	0.237	*Bartonella*	0.871	0.618
Christensenellaceae	0.329	0.085	*Bifidobacterium*	0.543	0.103
Coriobacteriaceae	0.557	0.069	*Blautia*	0.543	0.108
Cyanobiaceae	0.386	0.140	*Bombella*	0.343	0.040
Dysgonomonadaceae	0.500	0.091	*Burkholderia*	1.000	1.026
Enterobacteriaceae	1.000	0.699	*Carnimonas*	0.943	1.626
Erwiniaceae	1.000	4.551	*Catenibacterium*	0.486	0.016
Erysipelatoclostridiaceae	0.486	0.016	*Caulobacter*	0.700	0.237
Hafniaceae	0.571	0.103	*Chryseobacterium*	0.529	0.494
Halomonadaceae	1.000	1.963	*Citrobacter*	0.971	0.327
Lachnospiraceae	0.714	0.250	*Collinsella*	0.557	0.069
Lactobacillaceae	1.000	0.650	*Commensalibacter*	0.343	0.029
Leuconostocaceae	0.986	2.177	*Coprococcus*	0.257	0.025
Microbacteriaceae	0.929	1.737	*Dysgonomonas*	0.500	0.091
Moraxellaceae	0.986	1.251	*Enterobacter*	0.829	0.209
Morganellaceae	1.000	9.498	*Erwinia*	0.386	0.028
Neisseriaceae	0.771	0.281	*Faecalibacterium*	0.600	0.065
Nitrososphaeraceae	0.557	0.273	*Frischella*	0.343	0.030
Orbaceae	0.886	0.472	*Fructobacillus*	0.886	1.414
Peptostreptococcaceae	0.471	0.018	*Fusicatenibacter*	0.271	0.069
Pseudomonadaceae	0.871	0.115	*Gilliamella*	0.871	0.441
Rhizobiaceae	0.914	0.680	*Gluconacetobacter*	0.171	0.122
Ruminococcaceae	0.814	0.275	*Hafnia-Obesumbacterium*	0.571	0.103
Sphingobacteriaceae	0.243	0.028	*Halomonas*	0.971	0.337
Sphingomonadaceae	0.900	0.652	*Klebsiella*	0.414	0.163
Spiroplasmataceae	1.000	67.048	*Lactobacillus*	1.000	0.650
Staphylococcaceae	0.557	0.055	*Lactococcus*	0.929	1.071
Streptococcaceae	0.929	1.071	*Leifsonia*	0.929	1.737
Thiomicrospiraceae	0.514	0.265	*Leuconostoc*	0.457	0.154
Weeksellaceae	0.529	0.494	*Pantoea*	0.886	0.300
Xanthobacteraceae	0.329	0.180	*Pseudomonas*	0.871	0.115
Yersiniaceae	0.743	0.059	*Romboutsia*	0.471	0.018
			Roseburia	0.400	0.047
			Rosenbergiella	1.000	4.223
			Ruminococcus	0.557	0.074
			Serratia	0.743	0.059
			Snodgrassella	0.771	0.281
			Sphingobacterium	0.243	0.028
			Sphingomonas	0.900	0.652
			Spiroplasma	1.000	67.048
			Staphylococcus	0.557	0.055
			Subdoligranulum	0.614	0.136
			Synechococcus	0.386	0.140
			Thiomicrorhabdus	0.514	0.265
			Weissella	0.743	0.364

Table S6. Predicted score (ps) values of microbiota functionality score and percentage.

Sample	Amino Acids synthesis (ps)	Amino Acids synthesis (%)	Fatty Acid Biosynthesis (ps)	Fatty Acid Biosynthesis (%)	Metabolism of Aromatic Compounds (ps)	Metabolism of Aromatic Compounds (%)	Monosaccharides (ps)	Monosaccharides (%)	Nitrogen Metabolism (ps)	Nitrogen Metabolism (%)	Polysaccharides degradation (ps)	Polysaccharides degradation (%)	Protein degradation (ps)	Protein degradation (%)	Toxic compounds resistance (ps)	Toxic compounds resistance (%)	Vitamins (ps)	Vitamins (%)	Resistance to antibiotics (ps)	Resistance to antibiotics (%)
VoGB1	3300.13	**37.05**	312.23	**3.50**	3.19	**0.04**	803.34	**9.02**	408.81	**4.59**	917.31	**10.30**	1808.68	**20.30**	1326.75	**14.89**	12.57	**0.14**	15.30	**0.17**
VoGB10	3667.90	**37.84**	274.78	**2.84**	0.56	**0.01**	891.79	**9.20**	483.69	**4.99**	868.71	**8.96**	2188.97	**22.59**	1300.73	**13.42**	1.90	**0.02**	13.09	**0.14**
VoGB11	4646.69	**48.71**	21.87	**0.23**	0.45	**0.00**	1202.80	**12.61**	592.79	**6.21**	64.49	**0.68**	2913.93	**30.55**	93.77	**0.98**	0.75	**0.01**	1.01	**0.01**
VoGB12	3335.36	**51.47**	323.45	**4.99**	46.51	**0.72**	1060.65	**16.37**	189.64	**2.93**	198.22	**3.06**	1031.28	**15.91**	229.86	**3.55**	47.13	**0.73**	18.10	**0.28**
VoGB13	4131.66	**48.28**	183.59	**2.15**	2.23	**0.03**	1027.85	**12.01**	443.29	**5.18**	249.54	**2.92**	2097.05	**24.51**	411.54	**4.81**	6.69	**0.08**	3.46	**0.04**
VoGB14	4328.56	**48.91**	19.52	**0.22**	0.75	**0.01**	1121.29	**12.67**	550.97	**6.23**	53.22	**0.60**	2701.32	**30.52**	72.45	**0.82**	1.62	**0.02**	0.84	**0.01**
VoGB15	3240.86	**43.18**	130.56	**1.74**	1.60	**0.02**	819.30	**10.92**	411.89	**5.49**	388.32	**5.17**	1935.12	**25.79**	566.35	**7.55**	4.49	**0.06**	6.22	**0.08**
VoGB16	4347.16	**44.66**	121.77	**1.25**	0.86	**0.01**	1106.75	**11.37**	556.02	**5.71**	371.65	**3.82**	2668.69	**27.42**	552.81	**5.68**	2.14	**0.02**	5.73	**0.06**
VoGB17	4872.66	**49.03**	13.86	**0.14**	0.21	**0.00**	1263.30	**12.71**	620.97	**6.25**	40.16	**0.40**	3066.82	**30.86**	60.08	**0.60**	0.25	**0.00**	0.65	**0.01**
VoGB18	3332.89	**37.64**	262.11	**2.96**	1.02	**0.01**	811.62	**9.17**	434.29	**4.90**	811.33	**9.16**	1968.72	**22.23**	1219.28	**13.77**	1.81	**0.02**	11.99	**0.14**
VoGB19	2087.40	**40.25**	157.42	**3.04**	5.24	**0.10**	528.24	**10.19**	252.18	**4.86**	416.10	**8.02**	1120.91	**21.61**	604.26	**11.65**	7.27	**0.14**	6.87	**0.13**
VoGB2	4639.65	**49.05**	18.87	**0.20**	0.96	**0.01**	1204.21	**12.73**	588.93	**6.23**	47.39	**0.50**	2893.31	**30.59**	62.52	**0.66**	2.04	**0.02**	0.88	**0.01**
VoGB20	4449.28	**49.38**	7.26	**0.08**	0.48	**0.01**	1156.01	**12.83**	565.47	**6.28**	16.02	**0.18**	2793.16	**31.00**	20.38	**0.23**	0.95	**0.01**	0.37	**0.00**
VoGB21	3550.30	**48.08**	40.60	**0.55**	1.82	**0.02**	917.78	**12.43**	449.66	**6.09**	108.90	**1.47**	2165.44	**29.33**	142.71	**1.93**	4.42	**0.06**	1.89	**0.03**
VoGB22	4539.54	**49.38**	6.60	**0.07**	0.30	**0.00**	1179.59	**12.83**	576.95	**6.28**	15.98	**0.17**	2852.51	**31.03**	19.94	**0.22**	1.02	**0.01**	0.41	**0.00**
VoGB23	2063.91	**45.67**	120.27	**2.66**	7.01	**0.16**	515.99	**11.42**	239.48	**5.30**	282.28	**6.25**	962.48	**21.30**	298.70	**6.61**	23.19	**0.51**	6.37	**0.14**
VoGB24	2013.37	**48.38**	107.24	**2.58**	7.96	**0.19**	501.10	**12.04**	223.14	**5.36**	205.42	**4.94**	901.07	**21.65**	171.07	**4.11**	24.81	**0.60**	6.12	**0.15**
VoGB3	4706.84	**48.93**	17.43	**0.18**	0.43	**0.00**	1219.29	**12.68**	600.01	**6.24**	51.06	**0.53**	2951.28	**30.68**	70.35	**0.73**	1.24	**0.01**	0.98	**0.01**

VoGB4	3082.48	**49.74**	103.63	**1.67**	6.54	**0.11**	785.35	**12.67**	339.08	**5.47**	164.52	**2.65**	1574.86	**25.41**	107.74	**1.74**	26.27	**0.42**	6.70	**0.11**
VoGB5	4460.91	**49.52**	9.59	**0.11**	0.71	**0.01**	1156.25	**12.84**	565.77	**6.28**	19.32	**0.21**	2778.32	**30.84**	13.26	**0.15**	2.82	**0.03**	0.55	**0.01**
VoGB6	2839.27	**40.74**	201.37	**2.89**	5.94	**0.09**	699.75	**10.04**	356.56	**5.12**	571.02	**8.19**	1494.93	**21.45**	774.28	**11.11**	16.33	**0.23**	9.45	**0.14**
VoGB7	4021.45	**45.51**	97.19	**1.10**	1.50	**0.02**	1028.25	**11.64**	512.36	**5.80**	286.08	**3.24**	2460.30	**27.84**	422.40	**4.78**	2.47	**0.03**	4.48	**0.05**
VoGB8	1905.76	**49.82**	152.19	**3.98**	20.30	**0.53**	562.64	**14.71**	150.28	**3.93**	139.50	**3.65**	714.16	**18.67**	146.88	**3.84**	23.93	**0.63**	9.86	**0.26**
VoGB9	4768.81	**47.93**	40.03	**0.40**	0.20	**0.00**	1230.19	**12.36**	609.46	**6.13**	123.44	**1.24**	2990.62	**30.06**	185.55	**1.86**	0.26	**0.00**	1.85	**0.02**
VoGBa1	3827.82	**41.45**	200.85	**2.18**	1.70	**0.02**	945.09	**10.23**	494.38	**5.35**	616.99	**6.68**	2246.29	**24.33**	883.50	**9.57**	7.88	**0.09**	9.70	**0.11**
VoGBa2	3973.79	**41.60**	209.51	**2.19**	1.79	**0.02**	998.21	**10.45**	500.78	**5.24**	623.11	**6.52**	2321.06	**24.30**	905.75	**9.48**	8.44	**0.09**	10.25	**0.11**
VoGBa3	4211.57	**42.67**	164.38	**1.67**	0.35	**0.00**	1056.53	**10.70**	546.74	**5.54**	517.24	**5.24**	2586.85	**26.21**	778.79	**7.89**	0.36	**0.00**	7.67	**0.08**
VoGBa4	4706.53	**47.78**	43.94	**0.45**	0.28	**0.00**	1213.85	**12.32**	601.10	**6.10**	134.63	**1.37**	2945.96	**29.91**	201.14	**2.04**	0.59	**0.01**	2.03	**0.02**
VoQC1	4826.03	**49.00**	15.80	**0.16**	0.30	**0.00**	1251.97	**12.71**	613.98	**6.23**	45.31	**0.46**	3029.61	**30.76**	63.90	**0.65**	1.07	**0.01**	0.83	**0.01**
VoQC2	3698.67	**48.26**	108.58	**1.42**	4.62	**0.06**	1136.69	**14.83**	375.19	**4.90**	558.77	**7.29**	1521.19	**19.85**	79.03	**1.03**	144.84	**1.89**	36.85	**0.48**
VoQC3	4739.78	**48.76**	20.21	**0.21**	0.22	**0.00**	1227.28	**12.63**	604.41	**6.22**	60.76	**0.63**	2977.50	**30.63**	89.17	**0.92**	0.58	**0.01**	0.98	**0.01**
VoSB1	4866.26	**49.53**	2.40	**0.02**	0.21	**0.00**	1264.71	**12.87**	619.02	**6.30**	4.36	**0.04**	3063.86	**31.18**	3.07	**0.03**	0.79	**0.01**	0.21	**0.00**
VoSB10	4868.09	**49.53**	2.05	**0.02**	0.18	**0.00**	1264.93	**12.87**	619.39	**6.30**	2.73	**0.03**	3067.89	**31.21**	3.21	**0.03**	0.34	**0.00**	0.08	**0.00**
VoSB11	4894.38	**49.35**	6.99	**0.07**	0.24	**0.00**	1271.40	**12.82**	622.42	**6.28**	17.94	**0.18**	3078.88	**31.05**	23.81	**0.24**	0.82	**0.01**	0.38	**0.00**
VoSB12	4921.14	**49.43**	4.02	**0.04**	0.13	**0.00**	1277.92	**12.83**	626.64	**6.29**	9.69	**0.10**	3102.39	**31.16**	14.46	**0.15**	0.18	**0.00**	0.17	**0.00**
VoSB13	4652.98	**49.43**	21.94	**0.23**	1.38	**0.01**	1203.02	**12.78**	586.88	**6.24**	48.24	**0.51**	2853.96	**30.32**	36.47	**0.39**	6.37	**0.07**	1.20	**0.01**
VoSB14	3727.75	**44.80**	123.58	**1.49**	1.36	**0.02**	943.13	**11.34**	466.25	**5.60**	336.88	**4.05**	2215.02	**26.62**	496.58	**5.97**	3.96	**0.05**	5.43	**0.07**
VoSB15	4512.04	**49.25**	19.45	**0.21**	1.45	**0.02**	1170.89	**12.78**	571.08	**6.23**	43.47	**0.47**	2791.47	**30.47**	46.41	**0.51**	3.79	**0.04**	1.03	**0.01**
VoSB2	620.61	**49.84**	32.22	**2.59**	4.05	**0.32**	177.14	**14.23**	58.12	**4.67**	32.75	**2.63**	280.42	**22.52**	32.00	**2.57**	6.07	**0.49**	1.82	**0.15**
VoSB3	4564.73	**49.42**	10.59	**0.11**	0.26	**0.00**	1204.05	**13.04**	572.15	**6.19**	61.16	**0.66**	2796.52	**30.28**	7.67	**0.08**	15.91	**0.17**	3.95	**0.04**
VoSB4	1261.25	**47.25**	68.98	**2.58**	7.57	**0.28**	352.20	**13.19**	128.43	**4.81**	95.65	**3.58**	617.96	**23.15**	124.35	**4.66**	8.44	**0.32**	4.35	**0.16**
VoSB5	4848.05	**49.53**	2.44	**0.02**	0.18	**0.00**	1260.49	**12.88**	616.35	**6.30**	4.82	**0.05**	3051.74	**31.18**	3.19	**0.03**	0.95	**0.01**	0.22	**0.00**
VoSB6	3299.07	**46.09**	82.71	**1.16**	3.04	**0.04**	854.62	**11.94**	409.65	**5.72**	210.95	**2.95**	1982.14	**27.69**	307.79	**4.30**	3.99	**0.06**	4.19	**0.06**
VoSB7	4091.88	**49.63**	105.24	**1.28**	3.04	**0.04**	1031.09	**12.51**	468.45	**5.68**	165.00	**2.00**	2210.42	**26.81**	142.62	**1.73**	21.47	**0.26**	5.30	**0.06**
VoSB8	4815.19	**49.53**	2.98	**0.03**	0.28	**0.00**	1251.55	**12.87**	612.03	**6.30**	3.58	**0.04**	3030.96	**31.18**	4.11	**0.04**	0.50	**0.01**	0.10	**0.00**
VoSB9	4745.20	**49.51**	2.58	**0.03**	0.19	**0.00**	1233.47	**12.87**	603.68	**6.30**	6.09	**0.06**	2987.65	**31.17**	4.96	**0.05**	0.92	**0.01**	0.26	**0.00**
VoUC1	4882.10	**49.53**	3.89	**0.04**	0.50	**0.01**	1269.21	**12.88**	620.43	**6.29**	4.33	**0.04**	3070.19	**31.15**	4.69	**0.05**	0.62	**0.01**	0.20	**0.00**
VoUC10	4881.53	**49.54**	3.70	**0.04**	0.52	**0.01**	1269.20	**12.88**	620.40	**6.30**	3.76	**0.04**	3070.24	**31.16**	3.76	**0.04**	0.63	**0.01**	0.24	**0.00**
VoUC11	3962.96	**48.02**	102.99	**1.25**	11.32	**0.14**	1020.06	**12.36**	496.32	**6.01**	206.95	**2.51**	2191.47	**26.55**	243.80	**2.95**	13.82	**0.17**	3.47	**0.04**
VoUC2	4781.70	**49.53**	10.94	**0.11**	1.62	**0.02**	1240.15	**12.85**	608.82	**6.31**	18.96	**0.20**	2972.39	**30.79**	16.51	**0.17**	2.27	**0.02**	0.18	**0.00**
VoUC3	4826.73	**49.54**	3.59	**0.04**	0.41	**0.00**	1255.07	**12.88**	613.59	**6.30**	7.35	**0.08**	3031.47	**31.11**	4.08	**0.04**	1.48	**0.02**	0.30	**0.00**

VoUC4	4923.21	**49.53**	2.54	**0.03**	0.27	**0.00**	1278.42	**12.86**	626.83	**6.31**	3.98	**0.04**	3099.56	**31.19**	3.70	**0.04**	0.50	**0.00**	0.07	**0.00**
VoUC5	3124.21	**49.58**	35.22	**0.56**	4.12	**0.07**	819.67	**13.01**	382.61	**6.07**	59.24	**0.94**	1824.57	**28.95**	38.82	**0.62**	10.70	**0.17**	2.30	**0.04**
VoUC6	4942.63	**49.54**	1.82	**0.02**	0.11	**0.00**	1283.54	**12.86**	629.25	**6.31**	3.78	**0.04**	3113.84	**31.21**	2.00	**0.02**	0.69	**0.01**	0.16	**0.00**
VoUC7	4907.16	**49.53**	2.73	**0.03**	0.31	**0.00**	1274.42	**12.86**	624.87	**6.31**	4.50	**0.05**	3088.89	**31.18**	4.45	**0.04**	0.52	**0.01**	0.09	**0.00**
VoUC8	4587.06	**49.55**	15.40	**0.17**	2.02	**0.02**	1188.17	**12.84**	582.81	**6.30**	28.07	**0.30**	2828.09	**30.55**	21.05	**0.23**	3.84	**0.04**	0.39	**0.00**
VoUC9	4785.92	**49.53**	7.08	**0.07**	0.89	**0.01**	1245.81	**12.89**	606.40	**6.28**	9.74	**0.10**	2996.04	**31.01**	7.95	**0.08**	1.72	**0.02**	0.47	**0.00**
VoUCa1	2872.16	**46.66**	113.73	**1.85**	8.24	**0.13**	767.72	**12.47**	325.72	**5.29**	236.50	**3.84**	1532.68	**24.90**	269.05	**4.37**	20.47	**0.33**	8.67	**0.14**
VoUCa2	4400.37	**49.50**	7.71	**0.09**	0.08	**0.00**	1144.61	**12.88**	556.50	**6.26**	20.32	**0.23**	2742.55	**30.85**	12.25	**0.14**	3.80	**0.04**	0.98	**0.01**
VoUCa3	4469.79	**49.37**	12.27	**0.14**	0.30	**0.00**	1182.29	**13.06**	558.79	**6.17**	71.23	**0.79**	2725.03	**30.10**	10.51	**0.12**	18.22	**0.20**	4.56	**0.05**
VoUCa4	4813.27	**49.49**	9.04	**0.09**	0.45	**0.00**	1254.68	**12.90**	607.79	**6.25**	24.82	**0.26**	2998.28	**30.83**	12.03	**0.12**	5.04	**0.05**	1.23	**0.01**
VoUCb1	3121.24	**48.60**	50.50	**0.79**	3.85	**0.06**	825.75	**12.86**	376.28	**5.86**	104.59	**1.63**	1816.93	**28.29**	108.09	**1.68**	11.84	**0.18**	3.46	**0.05**
VoUCb2	4730.02	**49.50**	6.83	**0.07**	0.76	**0.01**	1228.57	**12.86**	601.57	**6.29**	13.78	**0.14**	2960.55	**30.98**	12.42	**0.13**	1.68	**0.02**	0.30	**0.00**
VoUCb3	4919.50	**49.52**	1.71	**0.02**	0.12	**0.00**	1279.09	**12.88**	626.25	**6.30**	6.23	**0.06**	3097.57	**31.18**	2.45	**0.02**	1.34	**0.01**	0.35	**0.00**
VoUCb4	2815.98	**49.43**	36.70	**0.64**	4.96	**0.09**	731.03	**12.83**	353.37	**6.20**	71.57	**1.26**	1616.78	**28.38**	54.93	**0.96**	10.14	**0.18**	1.64	**0.03**
VoUCb5	4695.90	**49.32**	18.81	**0.20**	0.35	**0.00**	1251.75	**13.15**	582.44	**6.12**	117.12	**1.23**	2803.78	**29.45**	12.91	**0.14**	30.27	**0.32**	7.48	**0.08**
VoUCb6	4353.22	**49.60**	17.68	**0.20**	0.87	**0.01**	1126.15	**12.83**	547.03	**6.23**	25.01	**0.28**	2679.54	**30.53**	24.24	**0.28**	3.19	**0.04**	0.53	**0.01**
VoUCb7	4897.66	**49.52**	3.22	**0.03**	0.25	**0.00**	1274.06	**12.88**	622.22	**6.29**	7.49	**0.08**	3078.62	**31.13**	4.11	**0.04**	1.56	**0.02**	0.37	**0.00**
VoUCb8	2795.52	**49.51**	13.61	**0.24**	1.69	**0.03**	733.28	**12.99**	348.03	**6.16**	17.06	**0.30**	1718.24	**30.43**	14.58	**0.26**	3.16	**0.06**	1.12	**0.02**
VoUCb9	4876.73	**49.56**	6.03	**0.06**	0.16	**0.00**	1265.50	**12.86**	618.04	**6.28**	5.37	**0.05**	3058.43	**31.08**	8.53	**0.09**	0.47	**0.00**	0.12	**0.00**

Table S7. Summary of alpha diversity comparisons across four sampling sites (San Ġwann, Imsida, Qawra, and Gudja), based on Faith's Phylogenetic Diversity (Faith PD), Observed Features (richness), and Pielou's Evenness (evenness). Statistical comparisons were conducted using the Kruskal–Wallis H test. Values represent test statistics (H), unadjusted *p*-values, and multiple testing–corrected *q*-values (Benjamini–Hochberg) for overall and pairwise group comparisons. *q*-values are not reported for overall comparisons.

Metric	Group 1	Group 2	H	*p*-value	q-value
**Pielou's Evenness**	All Groups		2.096409888	0.552639409	N/A
**Pielou's Evenness**	Gudja (n=28)	MaltaUni (n=24)	1.895216	0.168615	0.837847
**Pielou's Evenness**	Gudja (n=28)	Qawra (n=3)	0.040179	0.841132	0.898614
**Pielou's Evenness**	Gudja (n=28)	SanGwann (n=15)	0.016234	0.898614	0.898614
**Pielou's Evenness**	MaltaUni (n=24)	Qawra (n=3)	0.857143	0.354539	0.837847
**Pielou's Evenness**	MaltaUni (n=24)	SanGwann (n=15)	0.653333	0.418923	0.837847
**Pielou's Evenness**	Qawra (n=3)	SanGwann (n=15)	0.031579	0.858955	0.898614
**Faith's PD**	All Groups		7.812578	0.050048	N/A
**Faith's PD**	Gudja (n=28)	MaltaUni (n=24)	0.323787	0.569341	0.854011
**Faith's PD**	Gudja (n=28)	Qawra (n=3)	0.004464	0.946729	0.946729
**Faith's PD**	Gudja (n=28)	SanGwann (n=15)	5.25974	0.021824	0.065473
**Faith's PD**	MaltaUni (n=24)	Qawra (n=3)	0.02381	0.877371	0.946729
**Faith's PD**	MaltaUni (n=24)	SanGwann (n=15)	6.900833	0.008616	0.051693
**Faith's PD**	Qawra (n=3)	SanGwann (n=15)	1.547368	0.213524	0.427049
**Observed Features**	All Groups		5.346073	0.148140	N/A
**Observed Features**	Gudja (n=28)	MaltaUni (n=24)	2.882957	0.089522	0.268565
**Observed Features**	Gudja (n=28)	Qawra (n=3)	0.540179	0.462359	0.693538
**Observed Features**	Gudja (n=28)	SanGwann (n=15)	4.001763	0.045453	0.268565
**Observed Features**	MaltaUni (n=24)	Qawra (n=3)	0.072939	0.787105	0.787105
**Observed Features**	MaltaUni (n=24)	SanGwann (n=15)	0.725282	0.394417	0.693538
**Observed Features**	Qawra (n=3)	SanGwann (n=15)	0.284211	0.593955	0.712746

Table S8. Summary of beta diversity comparisons across four sampling sites (San Ġwann, Imsida, Qawra, and Gudja), based on Weighted UniFrac distances. Values represent PERMANOVA results including the overall pseudo-F statistic, p-value, and the results of pairwise comparisons between sites. Statistical significance was assessed using 999 permutations, with multiple testing correction applied to pairwise comparisons (q-values).

Group 1	Group 2	Sample size	Permutations	pseudo-F	p-value	q-value
**All Groups**		70	999	6.613597	0.001	N/A
**Gudja**	**Imsida**	52	999	17.848318	0.001	0.006
**Gudja**	**Qawra**	31	999	1.230089	0.301	0.501
**Gudja**	**SanĠwann**	43	999	9.759627	0.002	0.006
**Imsida**	**Qawra**	27	999	0.679334	0.492	0.5904
**Imsida**	**SanĠwann**	39	999	0.997124	0.334	0.501
**Qawra**	**SanĠwann**	18	999	0.253573	0.884	0.884

Table S9. Absolute abundance (qPCR-based quantification) of microbial pathogens commonly found in bees and detected in *Vespa orientalis* guts, alongside total bacterial (eubacterial) load. Samples were collected from four sites (San Ġwann, Imsida, Qawra and Gudja), and data are expressed as the log copy number of the target gene/cell/spore. 'undet.' indicates undetected pathogens, while the final row shows the detection frequency (%) of each pathogen across all analysed hornets.

Sampling site	*Total Bacteria*	N. ceranae	Serratia	C. bombi	N. apis	N. bombi	C. mellificae	L. passim	A. bombi	Listeria	Salmonella
**S. Ġwann**	7.91±0.59	2.27±0.71	0.71±0.81	5.86±4.10	undet.	undet.	undet.	undet.	undet.	undet.	undet.
**Imsida**	8.14±0.64	2.81±0.74	1.76±0.81	4.01±3.43	undet.	undet.	undet.	undet.	undet.	undet.	undet.
**Qawra**	8.14±1.06	3.41±1.38	0.50±0.87	6.68±1.36	undet.	undet.	undet.	undet.	undet.	undet.	undet.
**Gudja**	8.12±1.09	2.60±0.64	0.99±0.68	3.43±3.32	undet.	undet.	undet.	undet.	undet.	undet.	undet.
**Frequency**	-	**97.15%**	**71.43%**	**62.86%**	**0%**	**0%**	**0%**	**0%**	**0%**	**0%**	**0%**

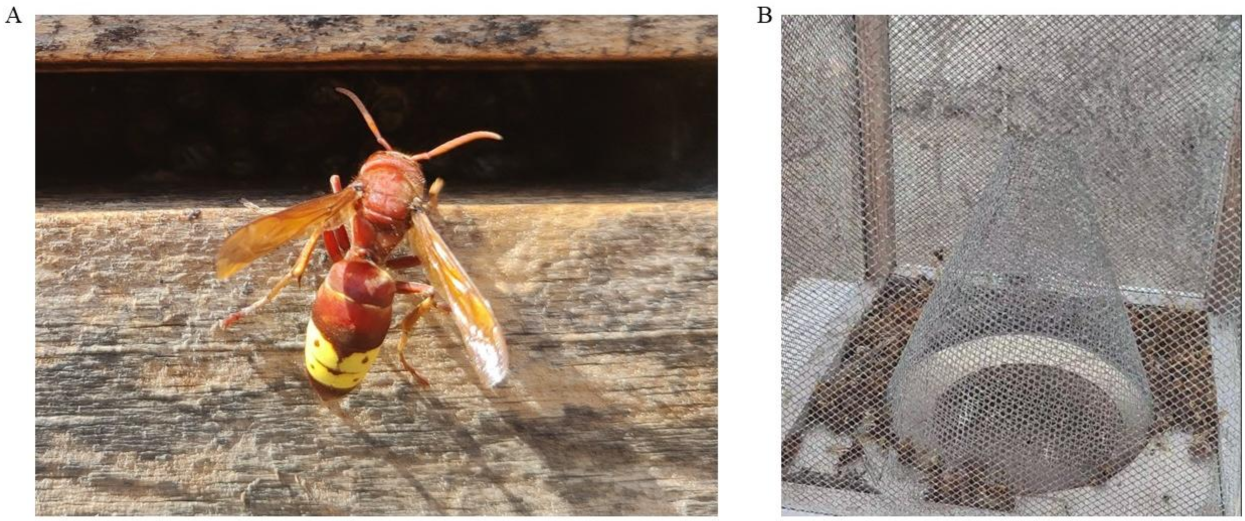

Figure S1. (A) *Vespa orientalis* specimen and (B) a *V. orientalis* trap placed near a honey bee colony, showing trapped hornets over a one-week period. Image credits: Jorge Spiteri.

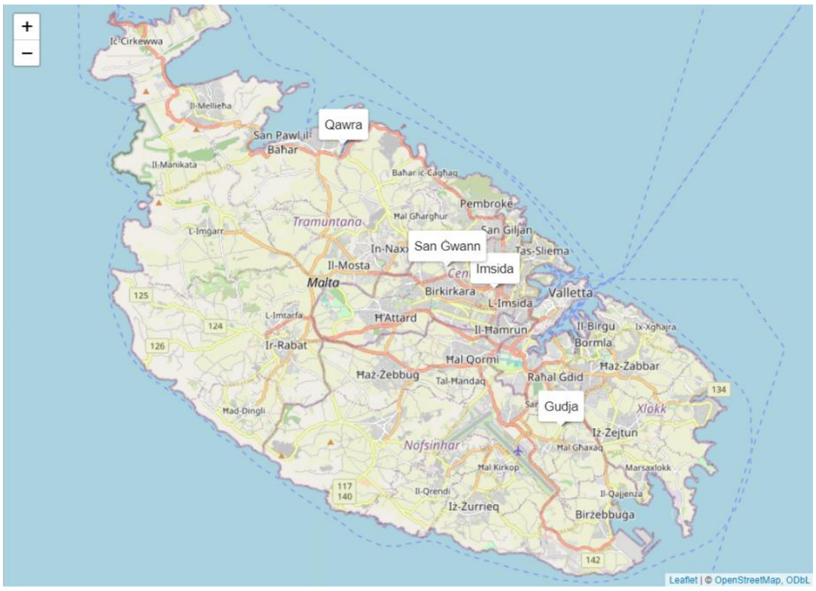

Figure S2. Map of the four sampling locations.

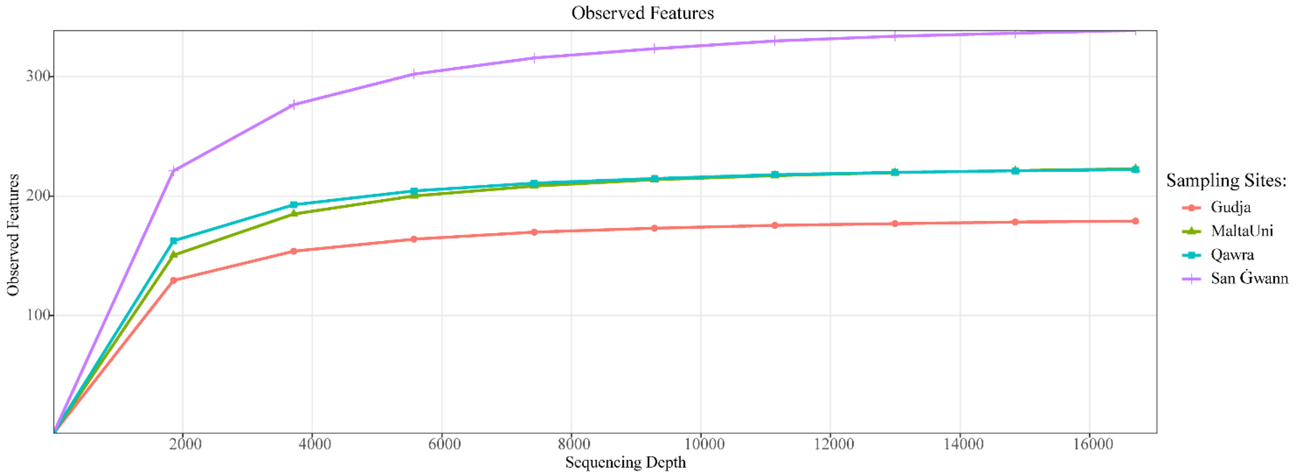

Figure S3. Alpha rarefaction curves showing the number of observed features in *Vespa orientalis* gut microbiota across five sampling sites. Rarefaction curves were generated from subsampled feature tables and plotted across sequencing depths up to 18,000 reads. Each curve represents the mean diversity per site. The flattening of curves at higher sequencing depths indicates that sequencing effort was sufficient to capture microbial richness within each group.

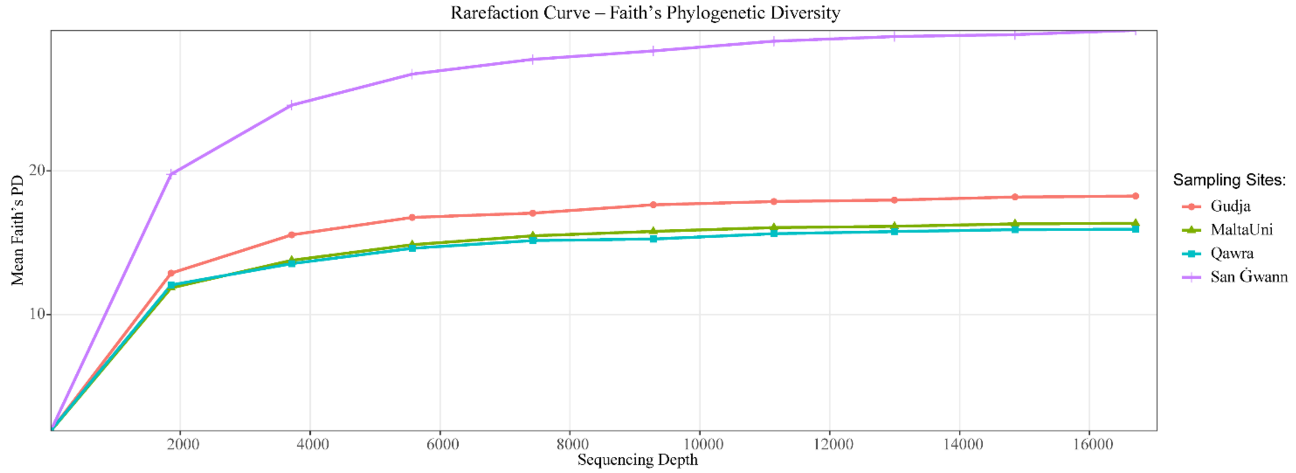

Figure S4. Alpha rarefaction curves showing Faith's Phylogenetic Diversity across gut microbiota from *Vespa orientalis* individuals collected at four sampling sites. Faith PD values were calculated at incremental sequencing depths up to 18,000 reads. Curves represent mean values per site, with error bars indicating variability among samples. The curves plateaued across all sites, indicating sufficient sequencing depth for phylogenetic diversity assessment.

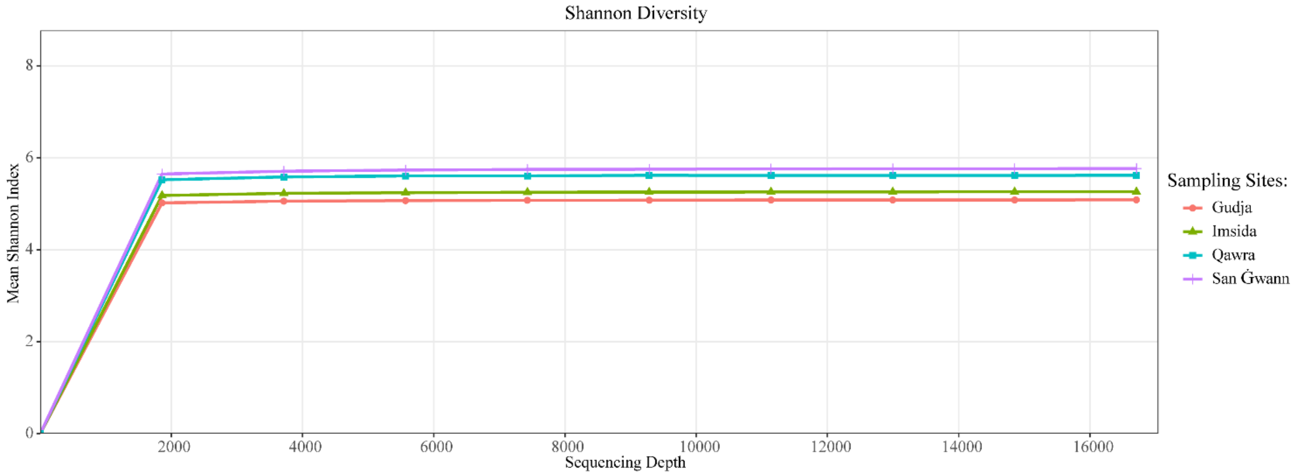

Figure S5. Alpha rarefaction curves showing Shannon diversity and number of samples retained per sequencing depth across gut microbiota from *Vespa orientalis* individuals collected at four sampling sites. Shannon index values were calculated at incremental sequencing depths up to 18,000 reads. Each line represents the mean diversity per site. The plateauing of curves indicates sufficient sequencing depth to capture microbial evenness.

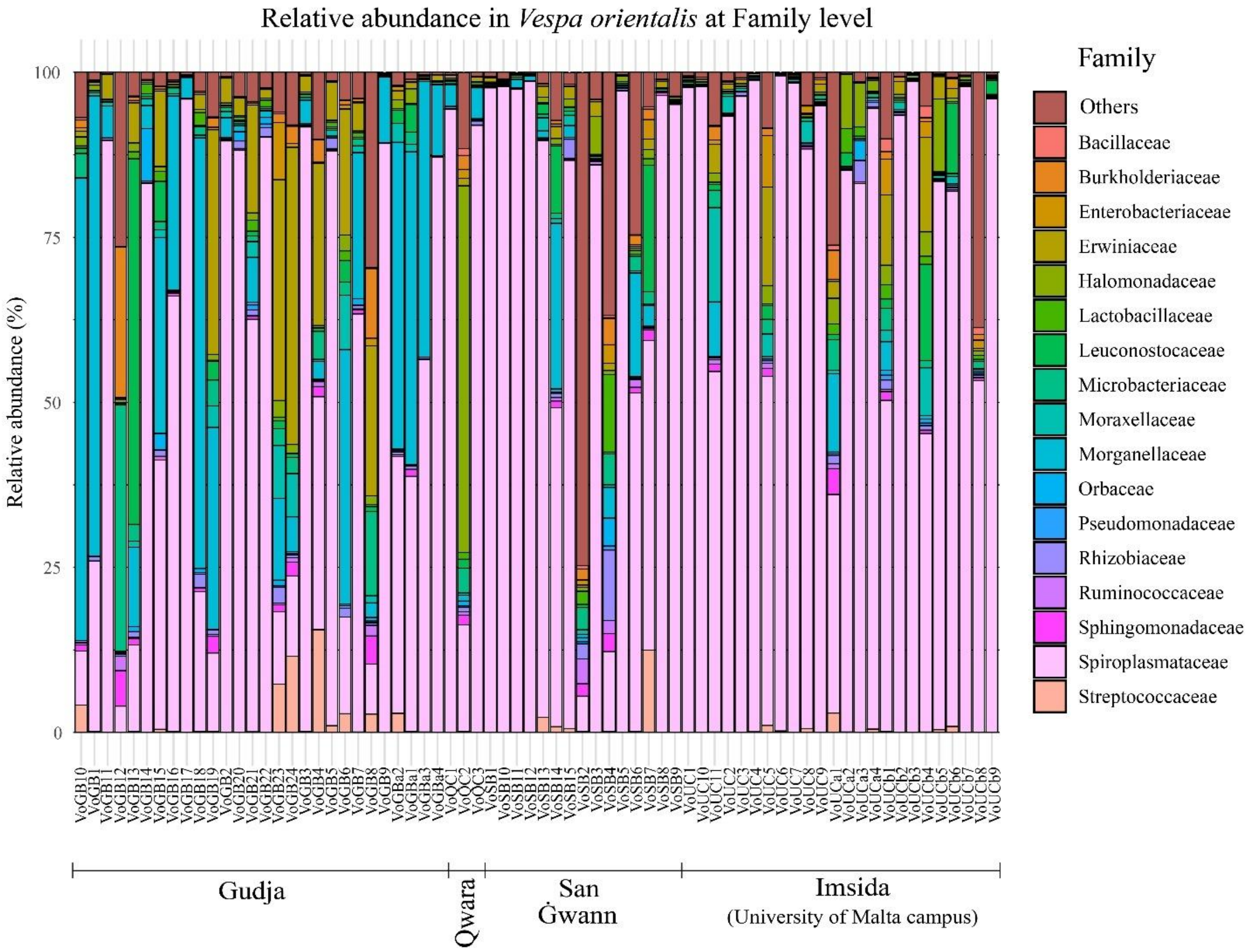

Figure S6. Relative abundance of *V. orientalis* gut microbiota at the family level. Families with a relative abundance of less than 1% in all samples are labelled as 'Other'.

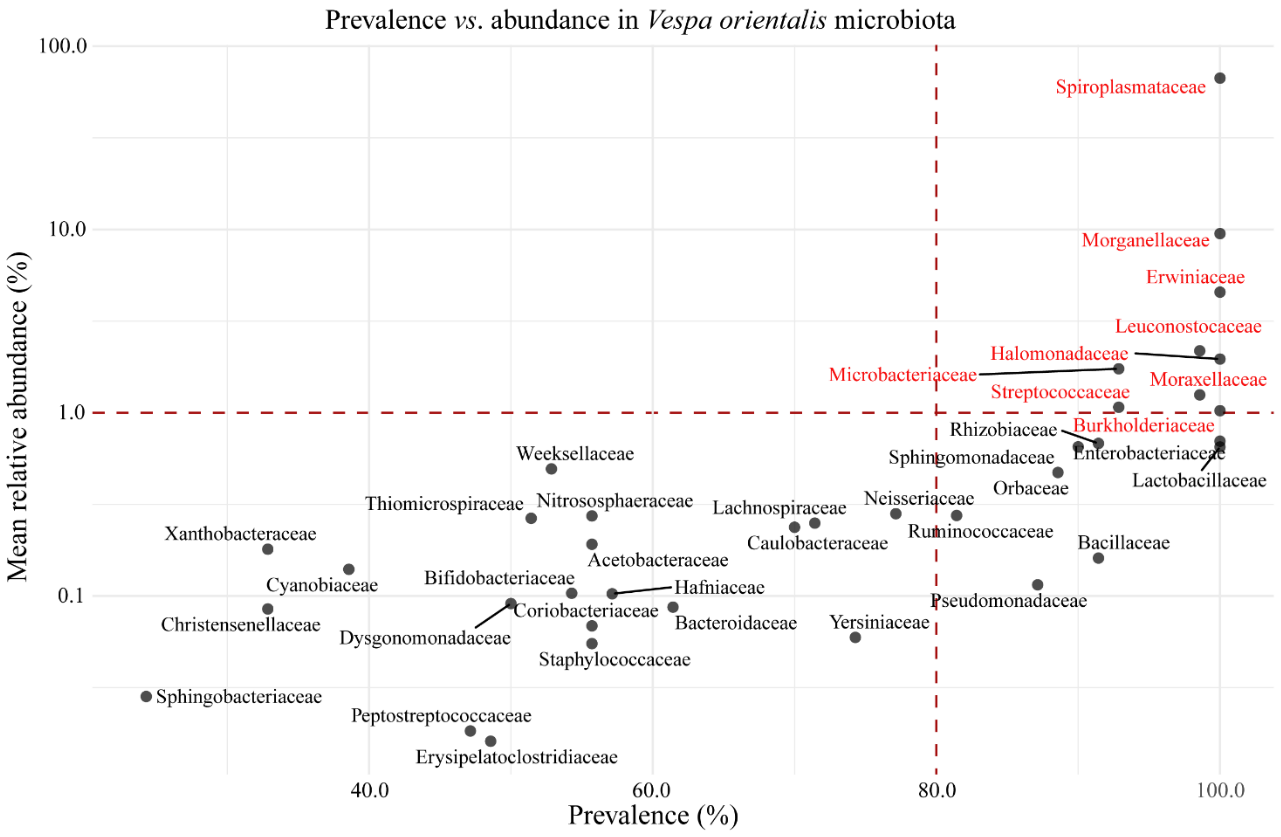

Figure S7. Scatter plot illustrating the prevalence and mean relative abundance of bacterial families within the *Vespa orientalis* gut microbiota. Each point represents a family, positioned according to its prevalence across samples and its average relative abundance. Red dashed lines indicate the selected cut-off values, while taxa highlighted in red represent the core microbiome of *V. orientalis*.

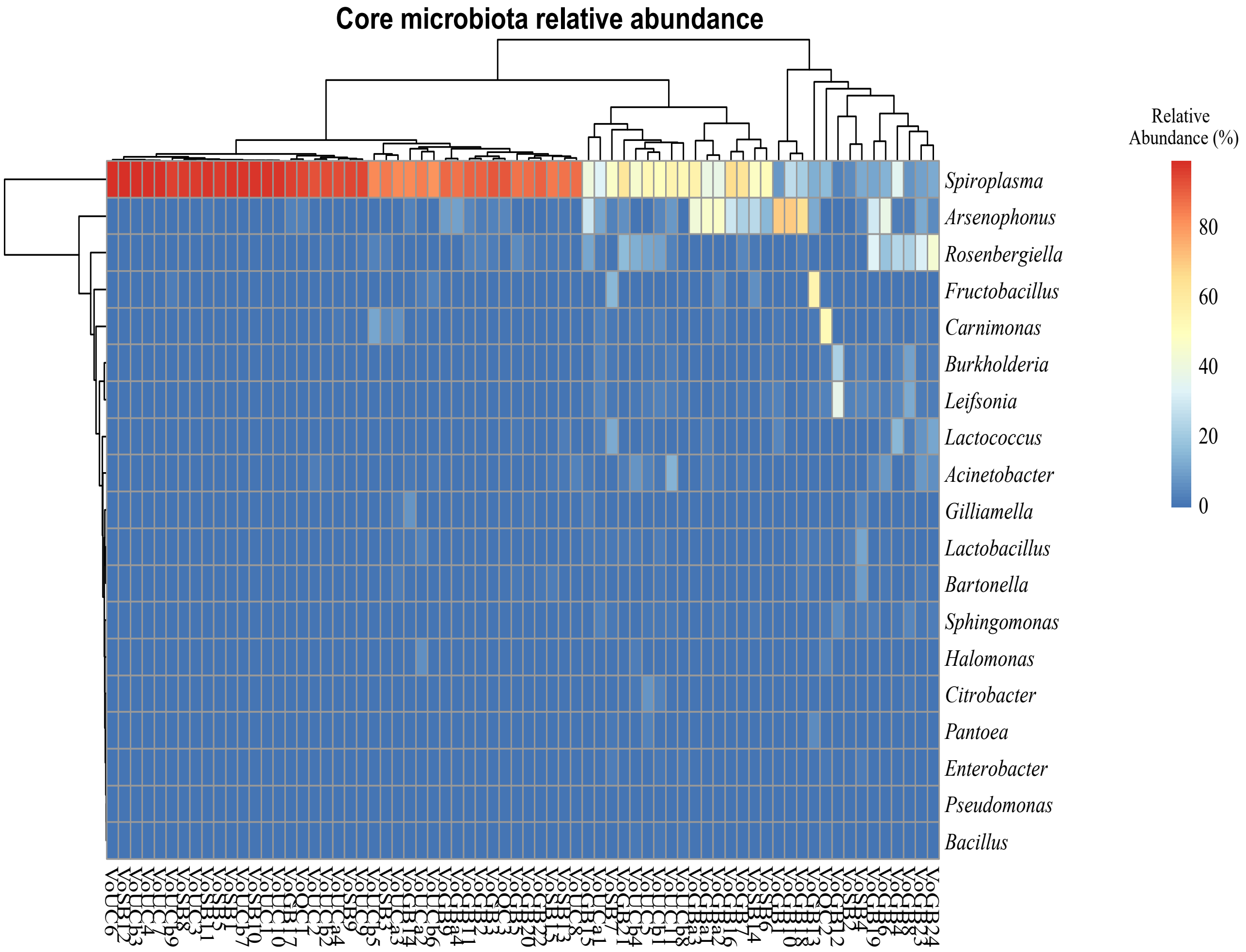

Figure S8. Heatmap showing the relative abundance of core microbiota taxa in *Vespa orientalis* gut samples. The colour gradient represents the relative abundance at bacterial genus level, ranging from blue (0%) to red (≥80%). Hierarchical clustering of samples (columns) and bacterial taxa (rows) is shown to highlight patterns of microbial community structure. Statistical differences in genus-level abundance across sites were assessed using the Kruskal–Wallis H test.

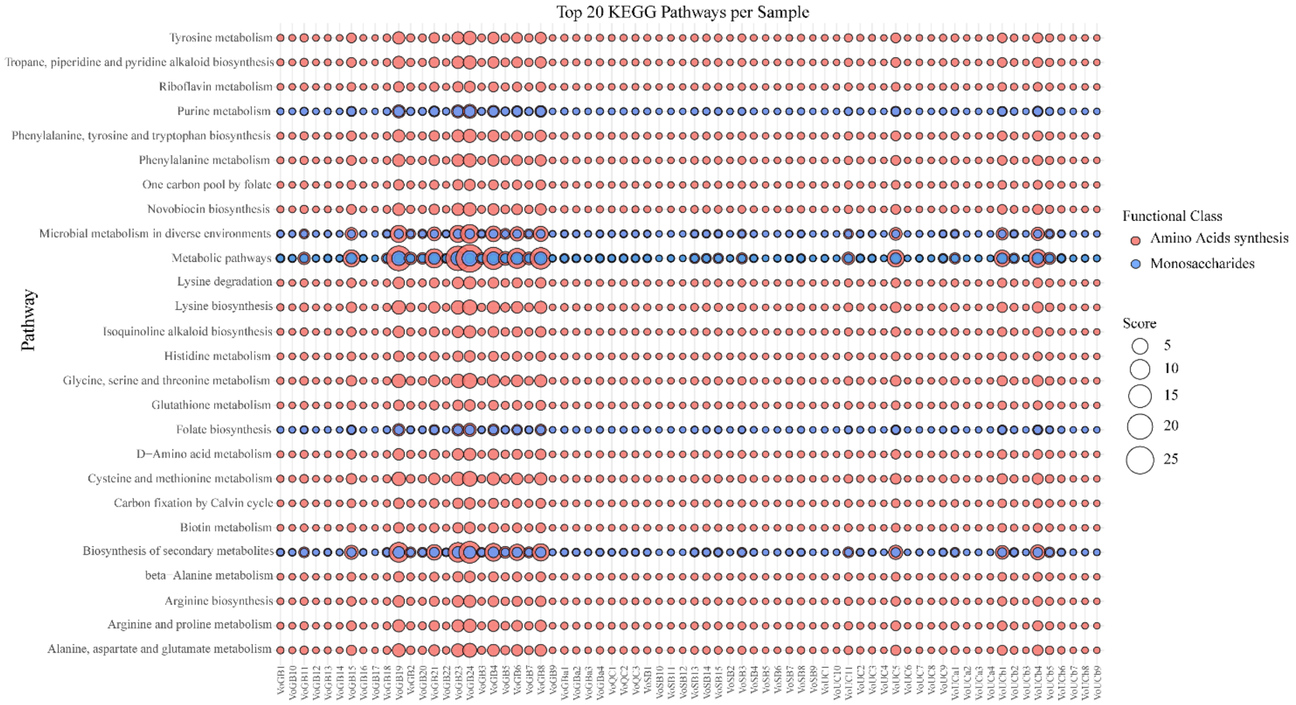

Figure S9. Bubble plot shows the top 20 KEGG most representative EC-number metabolisms per sample, differentiated by functional classes.

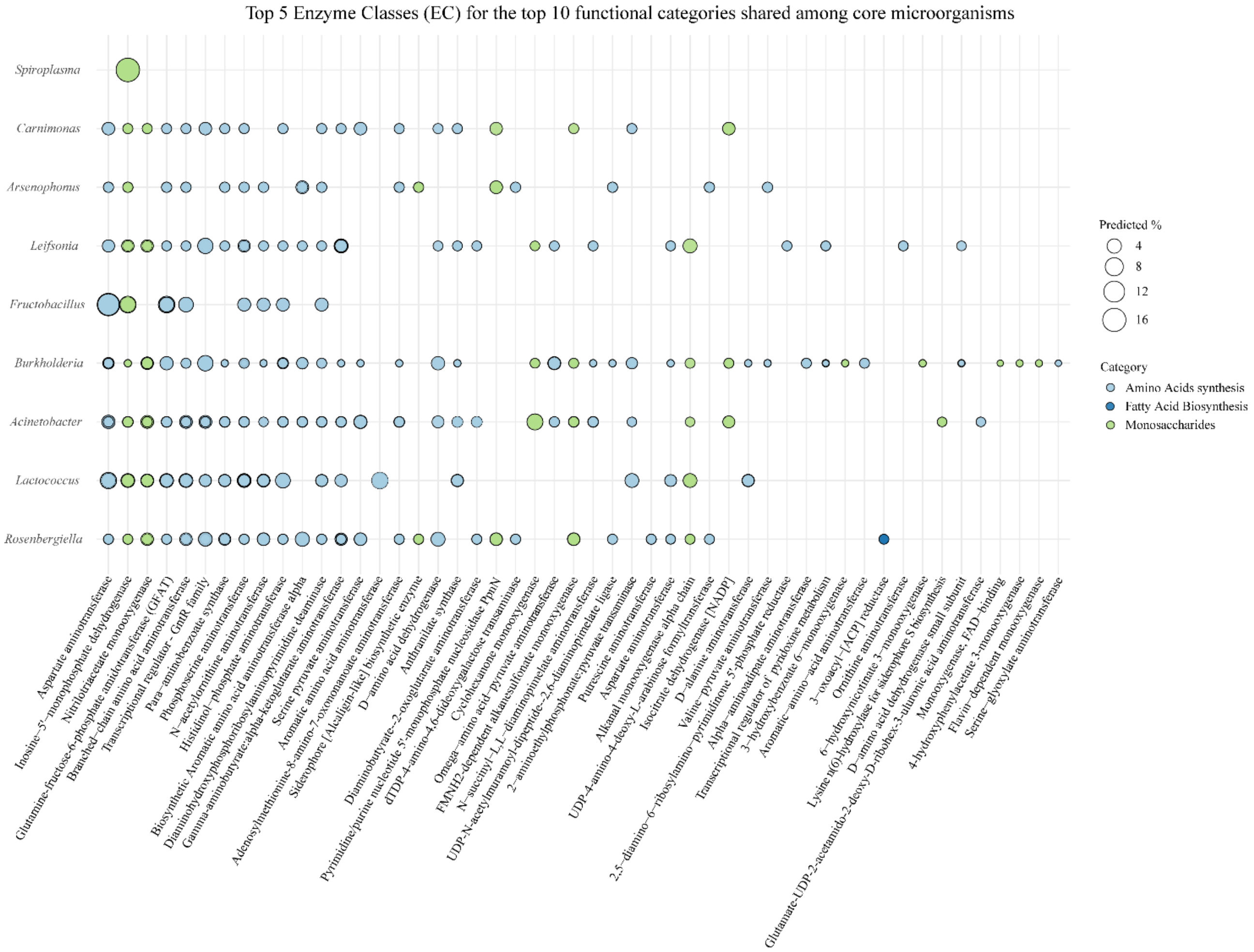

Figure S10. Bubble plot shows the top 5 KEGG most representative EC-number metabolisms for the *V. orientalis* microbial core taxa.

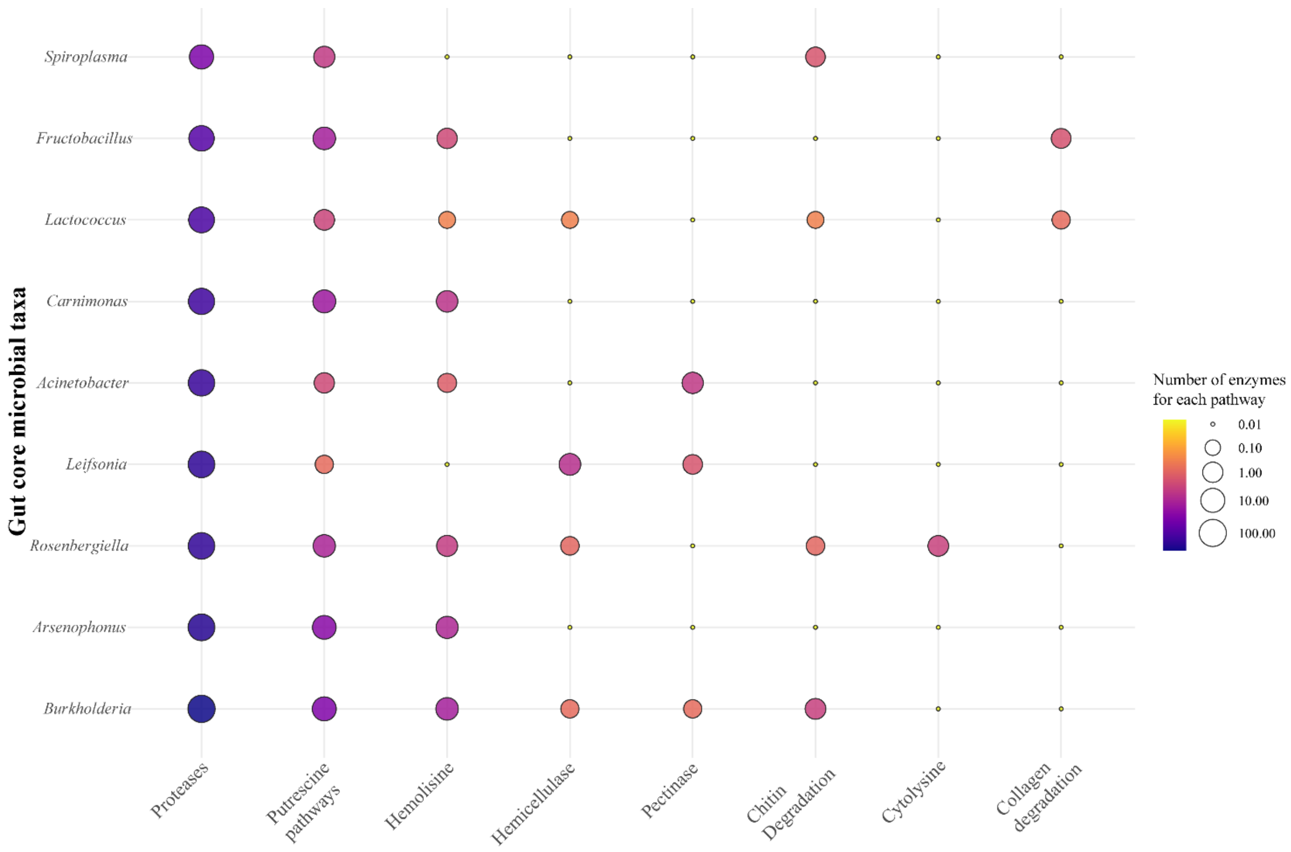

Figure S11. Number of predicted enzymes associated with animal tissue degradation functions (e.g., chitin degradation, collagen degradation, hemolysine, proteases, putrescine pathways, etc.) in the core gut microbial taxa identified. The bubble plot shows the functional potential of each genus based on the cumulative number of enzymes linked to each pathway.

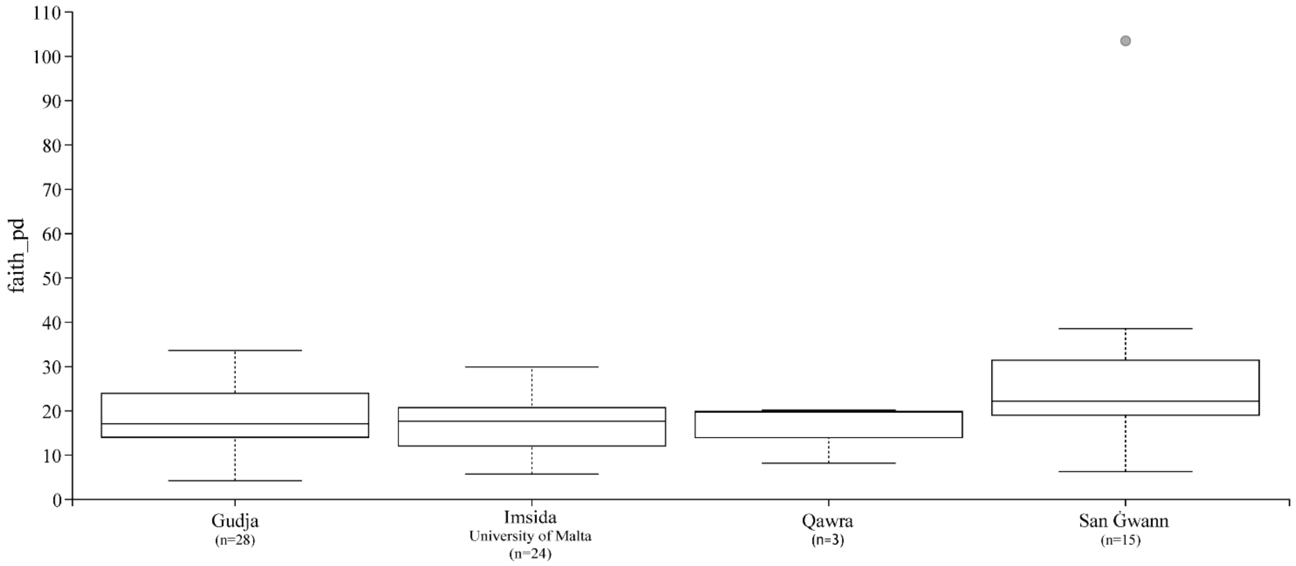

Figure S12. Boxplot showing Faith's Phylogenetic Diversity (PD) in *Vespa orientalis* gut microbiota across four sampling sites. Each box represents the interquartile range of PD values per site, with whiskers indicating variability and individual points representing outliers. No statistically significant differences in phylogenetic diversity were observed among sites (Kruskal–Wallis H test, $p > 0.05$).

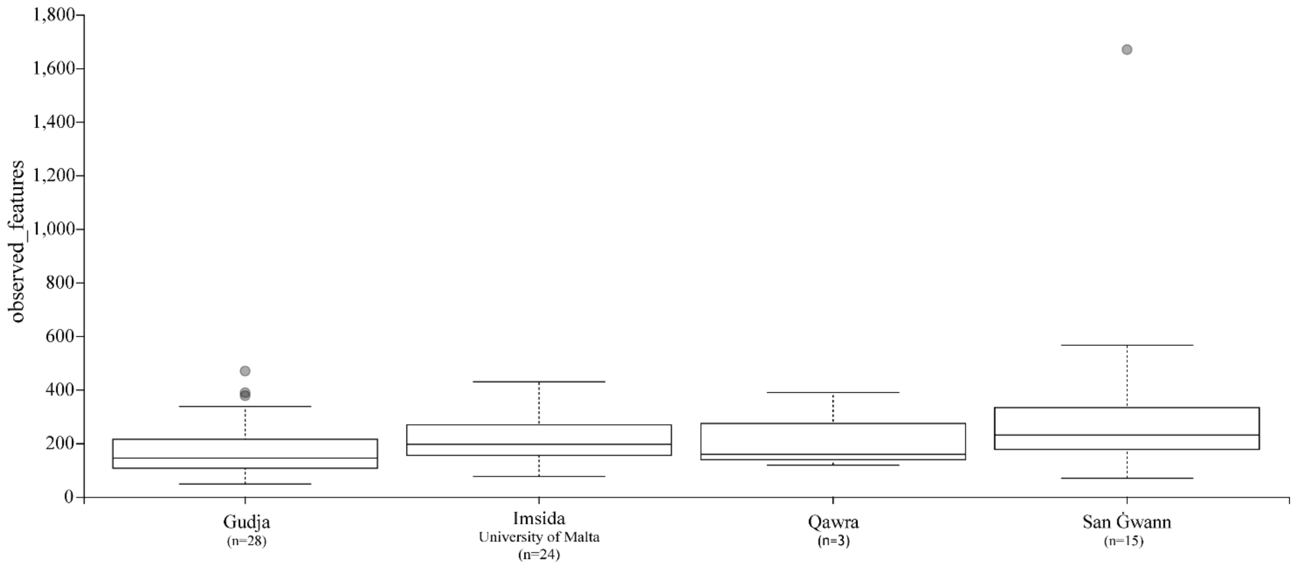

Figure S13. Boxplot showing the number of observed features (richness) in *Vespa orientalis* gut microbiota across the four sampling sites. Each point represents an individual hornet gut sample. No significant differences in richness were observed among sites (Kruskal–Wallis H test, $p > 0.05$).

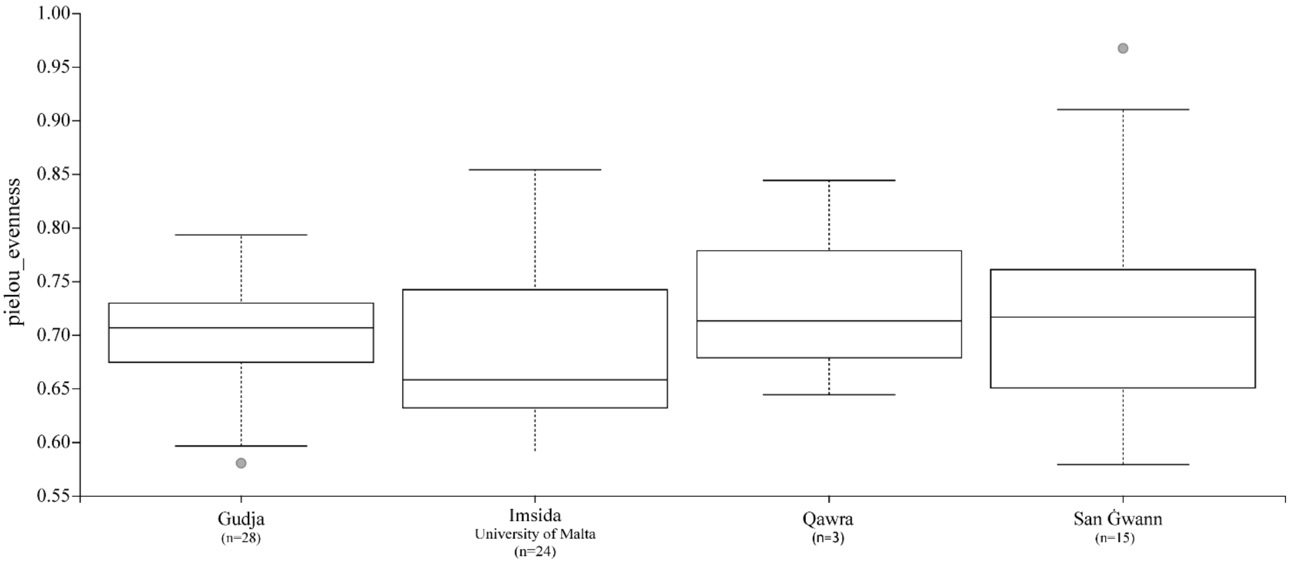

Figure S14. Boxplot showing Pielou's Evenness of *Vespa orientalis* gut microbiota across four sampling sites. Evenness reflects how uniformly individual sequences are distributed across taxa within each sample. Each box represents the interquartile range for each site, with whiskers indicating variability and points denoting outliers. No significant differences in evenness were detected among sites (Kruskal–Wallis H test, $p > 0.05$).

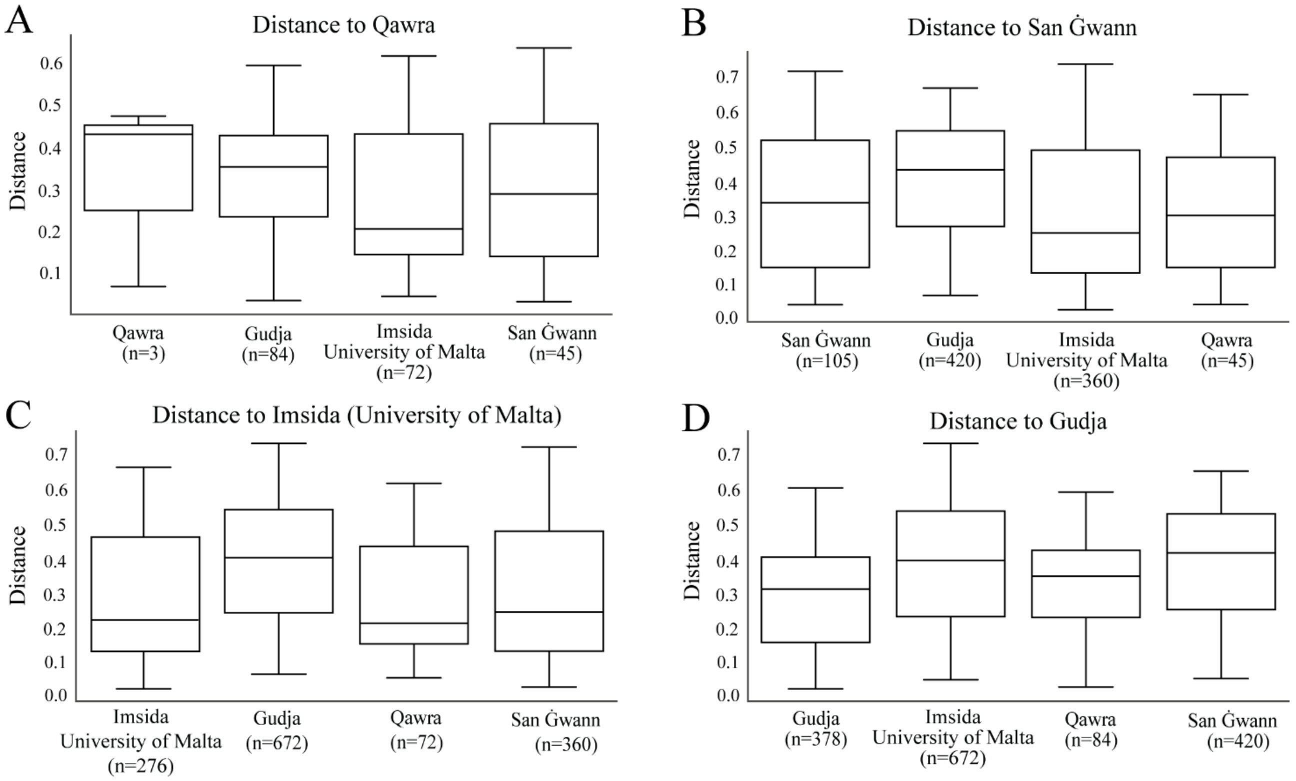

Figure S15. Boxplots showing beta diversity (Weighted UniFrac distances) to group centroids for hornets sampled from four sites. PERMANOVA revealed significant differences between Gudja and both Imsida and San Ġwann ($q < 0.05$) A) Qawra; B) San Ġwann; C) Imsida; D) Gudja.

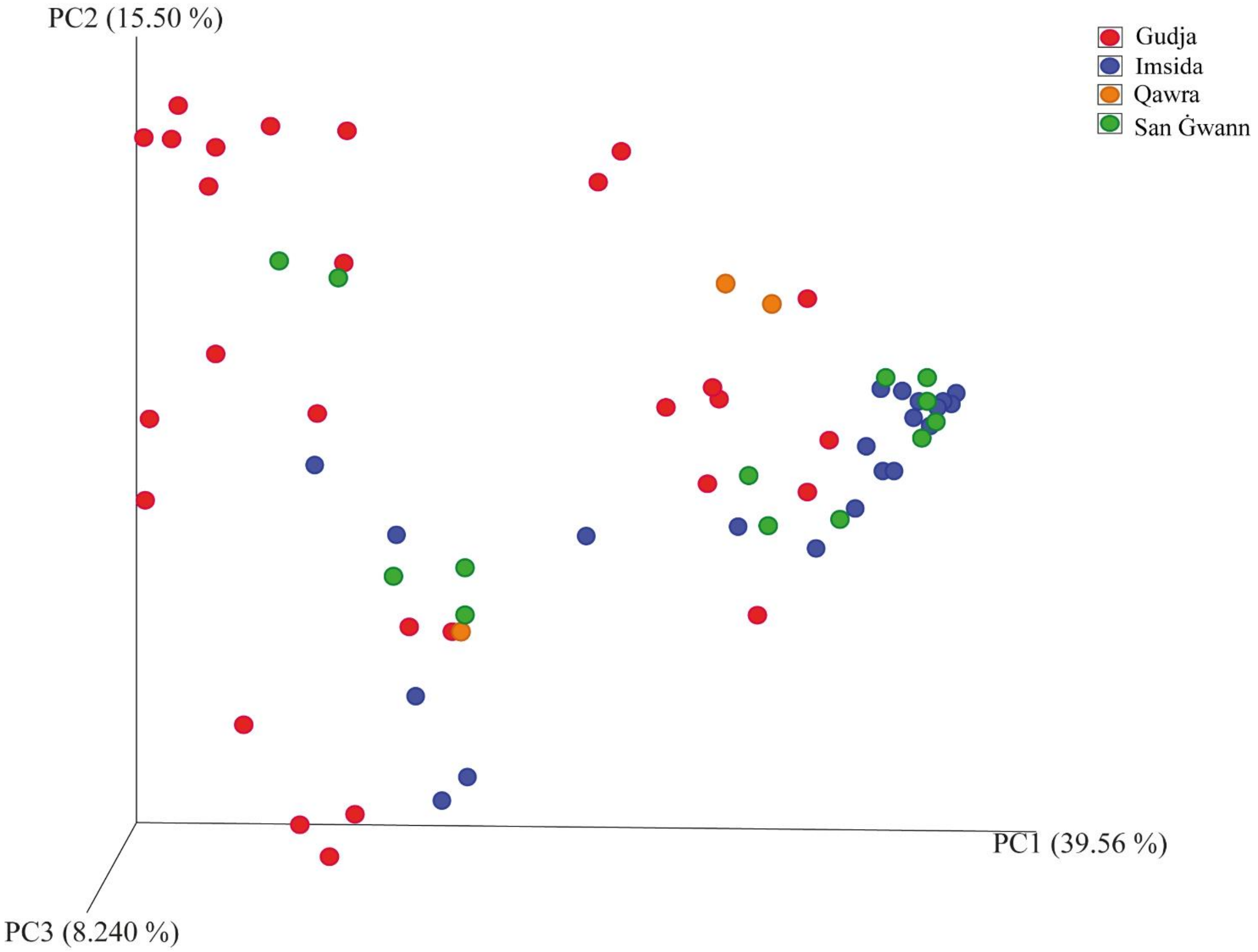

Figure S16. Principal Coordinates Analysis (PCoA) plot based on Weighted UniFrac distances, illustrating beta diversity of *Vespa orientalis* gut microbiota across four sampling sites. Each point represents an individual hornet gut sample. Colours indicate collection sites: Gudja (red), MaltaUni (blue), Qawra (orange), and San Ġwann (green). Axes represent the first three principal coordinates, explaining 39.56% (PC1), 15.50% (PC2), and 8.24% (PC3) of the variance, respectively. The dispersion of samples suggests high within-site variability in microbial community composition.

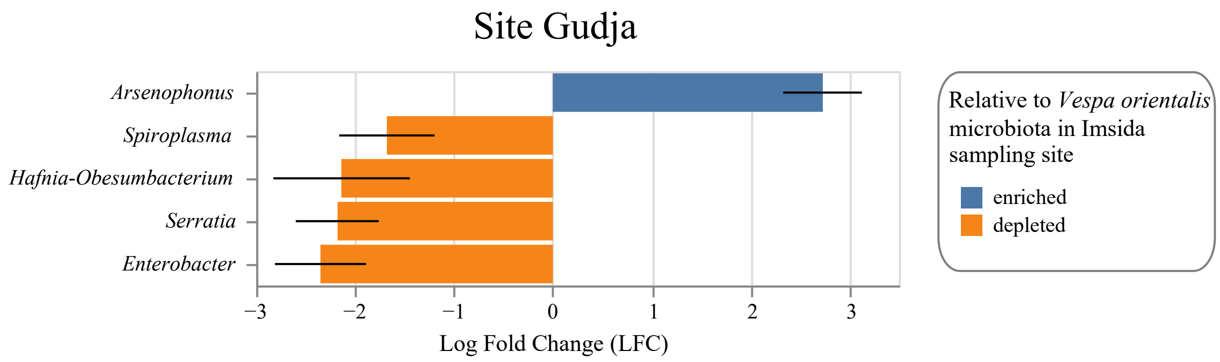

Figure S17. Differential abundance analysis of the microbiome relative abundance in hornets sampled at Gudja compared to hornets sampled at Imsida (University of Malta campus), highlighting which taxa are enriched (blue bars) or depleted (orange bars) in Gudja. Error bars represent the standard error of the log fold change (LFC) estimates.

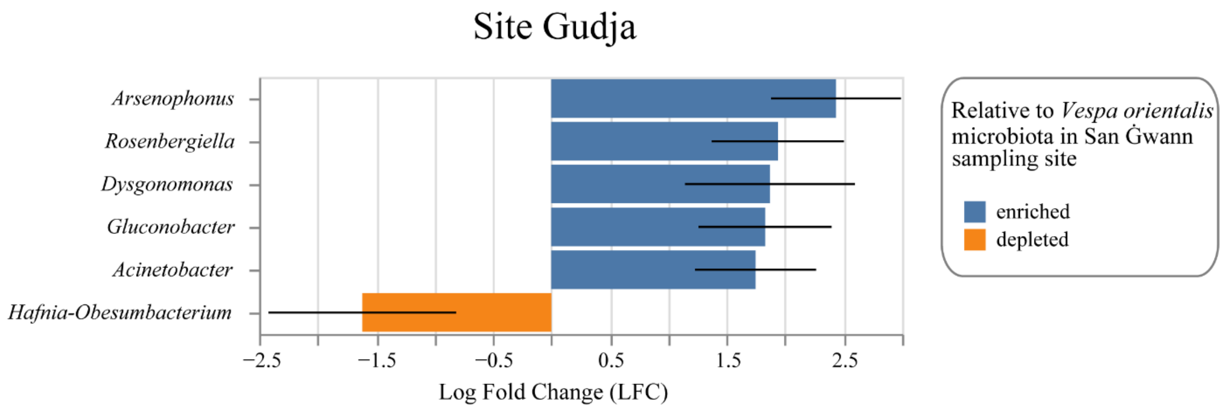

Figure S18. Differential abundance analysis of the microbiome relative abundance in hornets sampled at Gudja compared to hornets sampled at San Ġwann, highlighting which taxa are enriched (blue bars) or depleted (orange bars) in Gudja. Error bars represent the standard error of the log fold change (LFC) estimates.

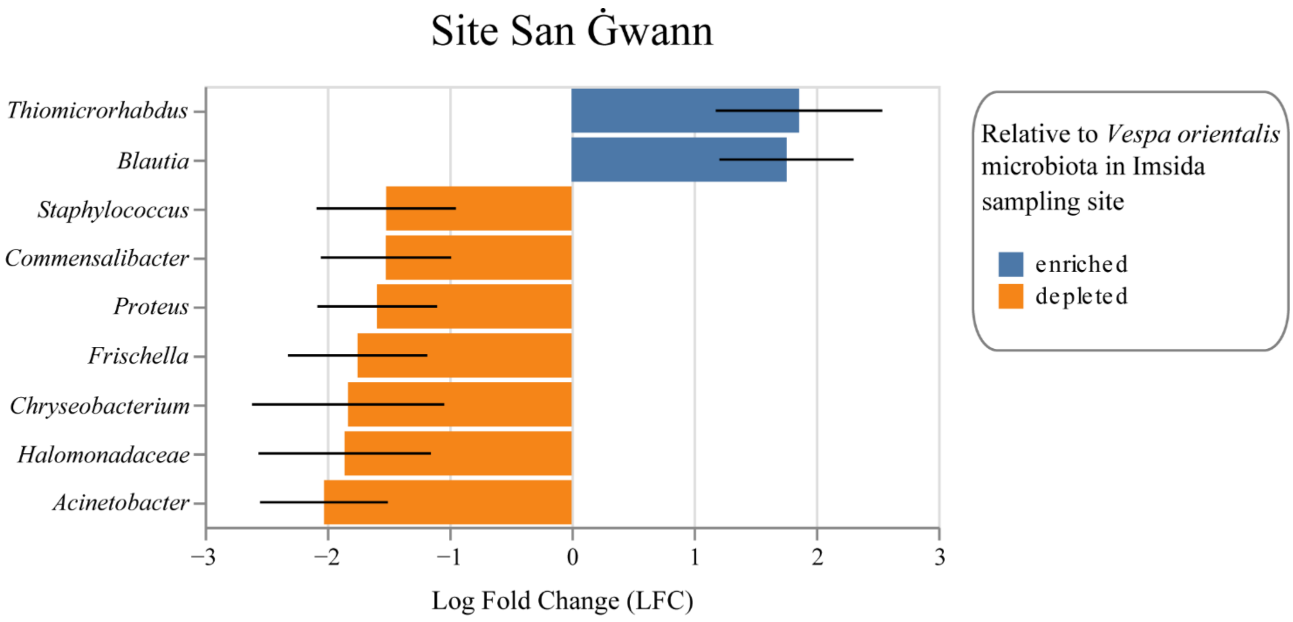

Figure S19. Differential abundance analysis of the microbiome relative abundance in hornets sampled at San Ġwann compared to hornets sampled at Imsida (University of Malta campus), highlighting which taxa are enriched (blue bars) or depleted (orange bars) in San Ġwann. Error bars represent the standard error of the log fold change (LFC) estimates.

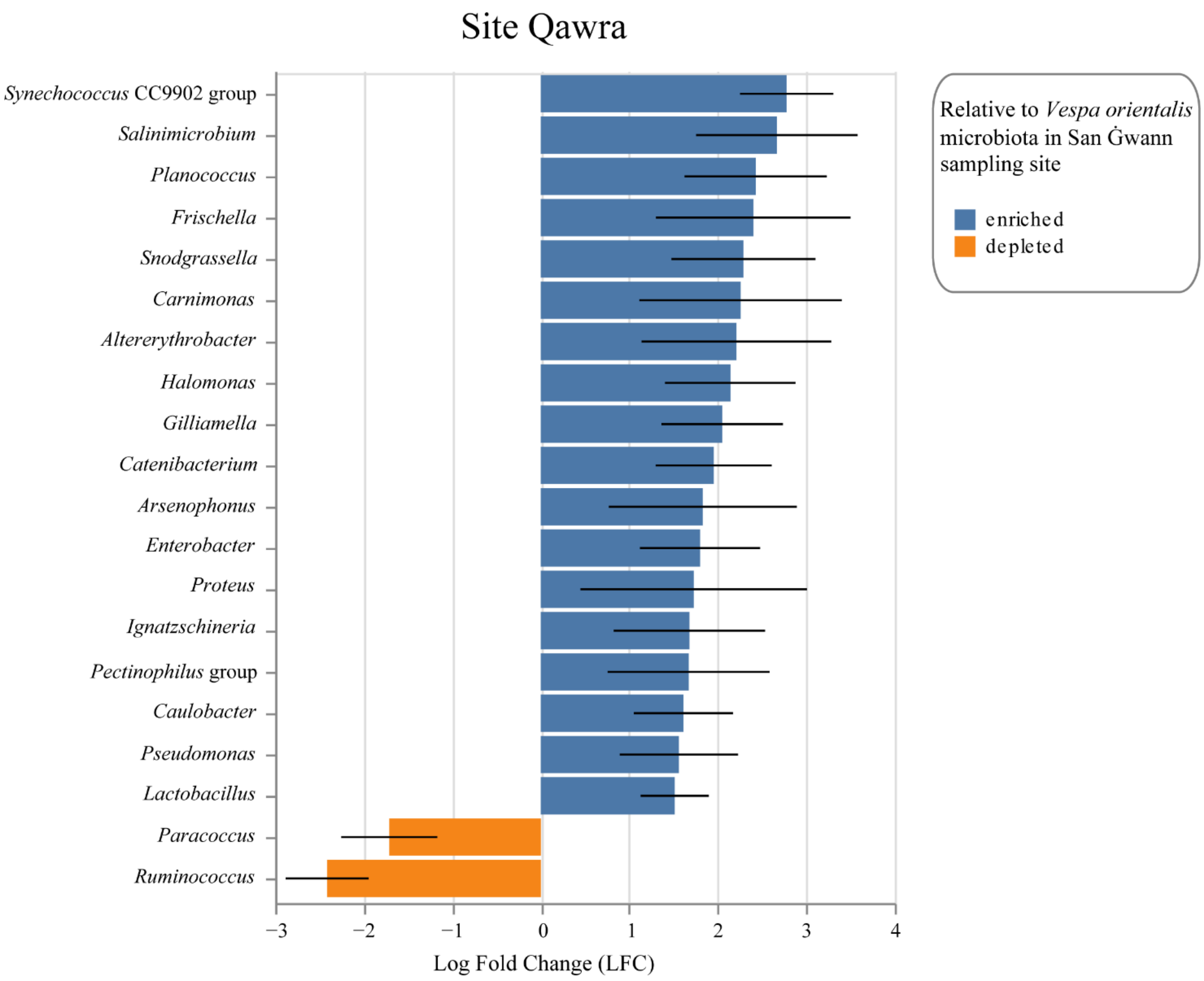

Figure S20. Differential abundance analysis of the microbiome relative abundance in hornets sampled at Qawra compared to hornets sampled at San Ġwann, highlighting which taxa are enriched (blue bars) or depleted (orange bars) in Qawra. Error bars represent the standard error of the log fold change (LFC) estimates.

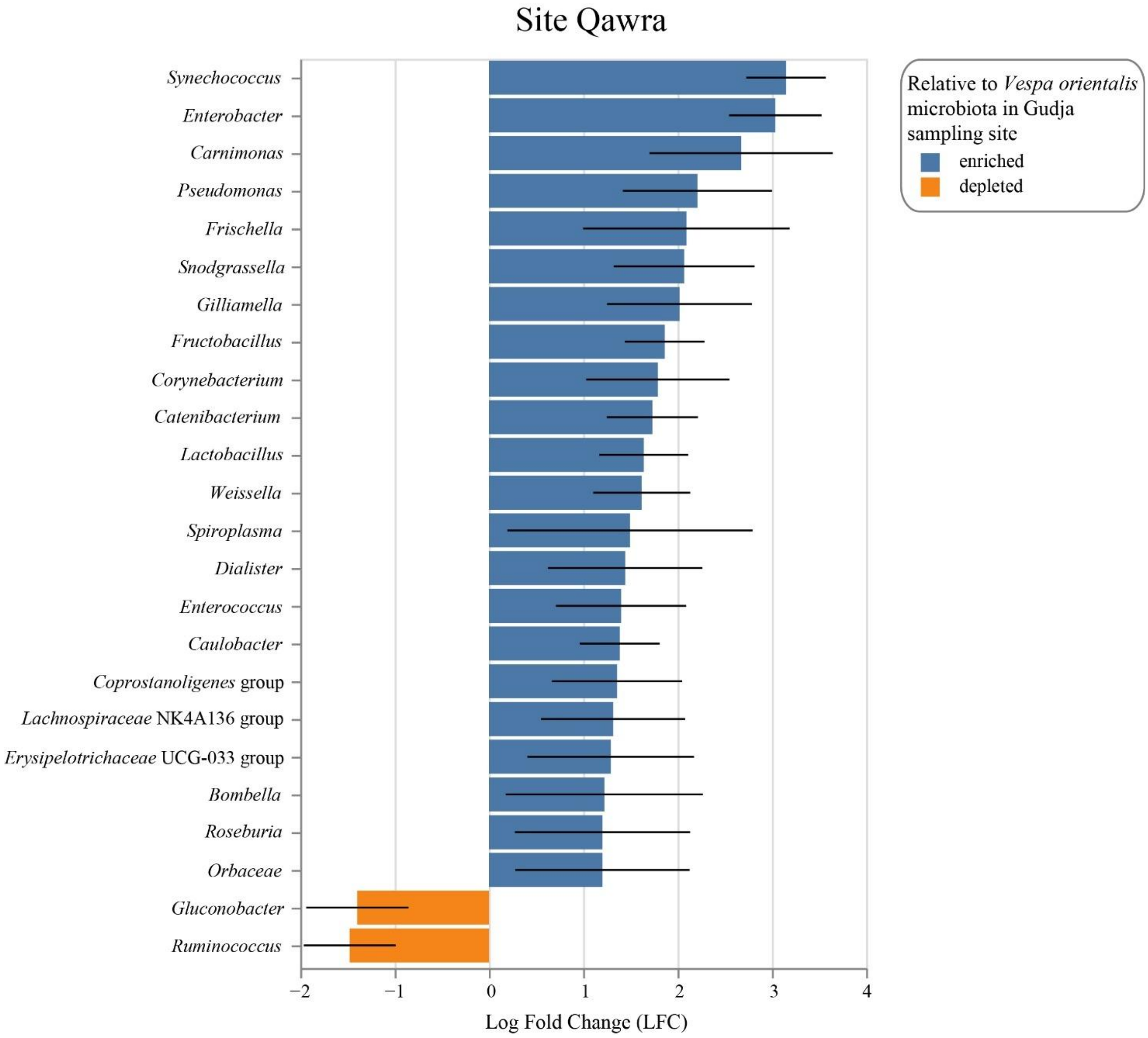

Figure S21. Differential abundance analysis of the microbiome relative abundance in hornets sampled at Qawra compared to hornets sampled at Gudja, highlighting which taxa are enriched (blue bars) or depleted (orange bars) in Qawra. Error bars represent the standard error of the log fold change (LFC) estimates.

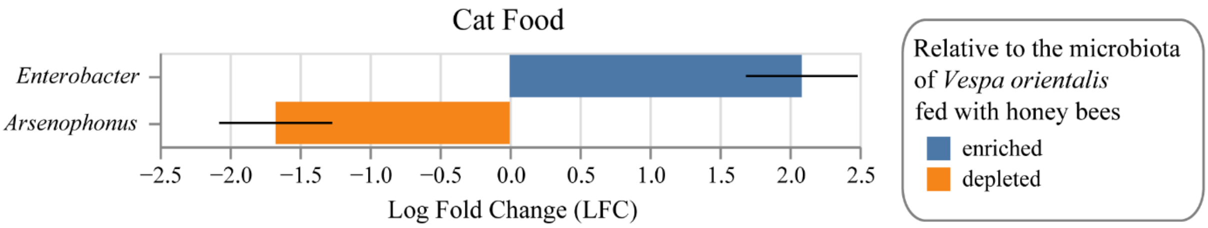

Figure S22. Differential abundance analysis comparing the gut microbiota of hornets feeding on cat food versus those feeding on honey bees. The plot highlights taxa that are enriched (blue bars) or depleted (orange bars) in hornets feeding on honey bees compared to those consuming cat food. Error bars represent the standard error of the log fold change (LFC) estimates.

Appendix 1

Malta Beekeepers Association *Vespa orientalis* Survey (2022 – 2024)

This appendix includes the original survey questions (in English and Maltese), followed by annual summaries of beekeeper responses (2022–2024). Key quantitative trends are presented in Supplementary Tables S1–S4 and Figures S1–S2. No inferential statistics were applied due to limited and uneven sample sizes.

A1.1 *Survey Questions and Answers*

Question 1: Sightings of *Vespa orientalis*

English:
"Have you ever observed Vespa orientalis?" (Photo attached – Yes/No)

Maltese:
"Ġieli rajt Bagħal taż-Żunżan – Vespa orientalis (ritratt anness)?"

Results Summary:

All responding beekeepers (100%) reported sightings of *V. orientalis* in their apiaries across all three years (2022–2024).

Question 2: Colony Losses

English Translation:

"Did you have any colony losses (more than 5–6 frames) because of the Oriental Hornet?" (Yes/No)

Maltese Original Text:

"Kellek telf ta' kolonji (iktar minn 5–6 tilari) minħabba l-Bagħal taż-Żunżan?"

Results Summary:

Survey responses showed fluctuations in reported colony losses over the three years. In 2022, 63.8% of beekeepers reported losses, followed by a sharp decline to 29.4% in 2023 and a resurgence to 59.5% in 2024. This trend suggests that 2023 was an anomalous year with reduced colony losses, possibly influenced by external factors (as explored in Question 7).

Appendix Table A1. Percentage of beekeepers reporting colony losses due to *Vespa orientalis* in 2022, 2023, and 2024.

Year	Total Respondents	Reported Losses (%)
**2022**	58	63.8%
**2023**	34	29.4%
**2024**	37	59.5%

Question 3: Months of Honey bee Losses

English Translation:

"In which month(s) did you notice honey bee losses due to the Oriental Hornet? Select as many as apply."

Maltese Original Text:

"F'liema xahar innutajt telf ta' naħal minħabba l-Bagħal taż-Żunżan? Agħżel kemm hemm bżonn."

Results Summary:

Beekeepers reported peak honey bee losses between July and October, with the highest losses occurring in September in all three years.

Appendix Table A2. Peak months of honey bee losses based on beekeeper responses.

Year	Peak Loss Months (Most Reported)
2022	August – September
2023	September – October
2024	July – September

Question 4: Description of Colony Loss

English Translation:

"How would you describe this bee colony loss? Select all that apply."

Maltese Original Text:

"Kif tiddeskrivieħ dan it-telf ta' naħal? Agħżel kemm hemm bżonn."

Results Summary:

Thematic analysis of beekeeper responses revealed several recurring issues affecting bee colonies. The most reported problem was wax moth infestation (25 occurrences), indicating that weakened or abandoned hives are highly vulnerable to pests. Beekeepers also frequently found their hives completely empty (19 occurrences), with some describing total apiary abandonment (11 occurrences). Additionally, unsuccessful queen replacement attempts were noted in 15 cases, suggesting colony instability. Bee losses were confirmed in 11 instances, while hornet attacks (eight reports) and food shortages were less commonly mentioned but still contributed to colony stress. These findings indicate that colony decline is influenced by multiple factors, including pest infestations, unsuccessful queen replacements, and predatory threats. The interconnection between these elements suggests that further research is needed to determine the root causes of colony instability and loss.

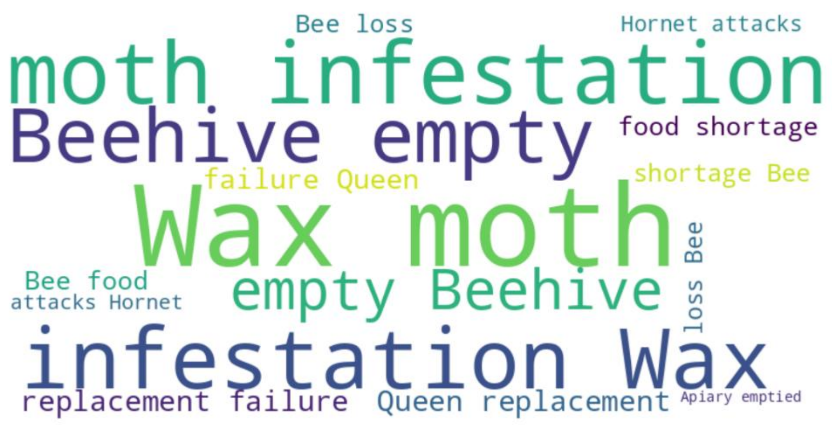

Appendix Figure A1. A word cloud depicting the most frequently reported issues affecting bee colonies in apiaries attacked by *Vespa orientalis*.

Question 5: Interventions Against *Vespa orientalis*

English Translation:

"Did you undertake any intervention(s) against the Oriental Hornet?" (Yes/No, plus optional free text to describe the intervention)

Maltese Original Text:

"Agħmilt xi interventi kontra l-Bagħal taż-Żunżan?"

Results Summary:

Beekeepers implemented various control measures to mitigate the impact of *Vespa orientalis*, including trapping queens and/or drones and manual removal of hornets. Intervention rates fluctuated over the three-year period. In 2022, 48 beekeepers (82.8%) reported taking action against the hornet. However, in 2023, intervention rates declined, with only 25 beekeepers (73.5%) engaging in control measures. A slight increase was observed in 2024, with 28 beekeepers (75.7%) reporting intervention efforts. This decline in intervention rates over time may suggest reduced beekeeper engagement, changes in hornet activity, or shifting perceptions of intervention effectiveness.

Appendix Table A3. Beekeeper intervention efforts against Vespa orientalis (2022–2024).

Year	Reported Intervention	Percentage of Respondents (%)
**2022**	48	82.8%
**2023**	25	73.5%
**2024**	28	75.7%

Question 6: Effectiveness of Interventions

English Translation:

"How effective do you think this intervention was?" (Score from *Ineffective* to *Very Effective*)

Maltese Original Text:

"Kemm taħseb li kien effettiv dan l-intervent?"

Results Summary:

Assessments of intervention effectiveness varied across the years. In 2023, 18 beekeepers (52.9%) considered their efforts effective, while 4 (11.8%) reported complete failure. By 2024, only 16 beekeepers (43.2%) found interventions successful, while those reporting failed control measures increased to 7 (18.9%).

Appendix Table A4. Perceived effectiveness of interventions against *Vespa orientalis*, based on beekeeper responses.

Year	Total Respondents	Reported Effectiveness (%)	Reported Failure (%)
**2023**	34	18 (52.9%)	4 (11.8%)
**2024**	37	16 (43.2%)	7 (18.9%)

Question 7: External Factors Affecting Hornet Populations

(Added in 2023 due to the observed anomalous decrease in hornet populations that year.)

English Translation:

"Do you think that, apart from your intervention, there was anything else that may have negatively affected Vespa orientalis in 2023? Select all that apply."

Maltese Original Text:

"Taħseb li, apparti l-intervent tiegħek, kien hemm xi ħaġa oħra li jaf affettwat ħażin lill-Bagħal taż-Żunżan fl-2023? Agħżel kemm hemm bżonn."

Results Summary:
Factors impacting bee populations. The most frequently reported issue was prolonged cold weather (17 occurrences), which may have delayed colony development and food availability. Limited access to food was another significant challenge (16 occurrences), with beekeepers noting that bees struggled to sustain themselves. Additionally, 13 responses indicated that human intervention, including efforts to control hornet populations, played a role in shaping bee activity. While the impact of extreme weather was less frequently mentioned, five respondents reported that the July heatwave had consequences on colony conditions. Unstable weather patterns were noted once, indicating that beekeepers may perceive seasonal variability as an emerging concern.

Overall, these results suggest that climatic conditions, food scarcity, and external pressures from both human and predator activity are the primary factors influencing beekeeping outcomes. Further research is needed to assess long-term trends and the interplay between these environmental stressors.

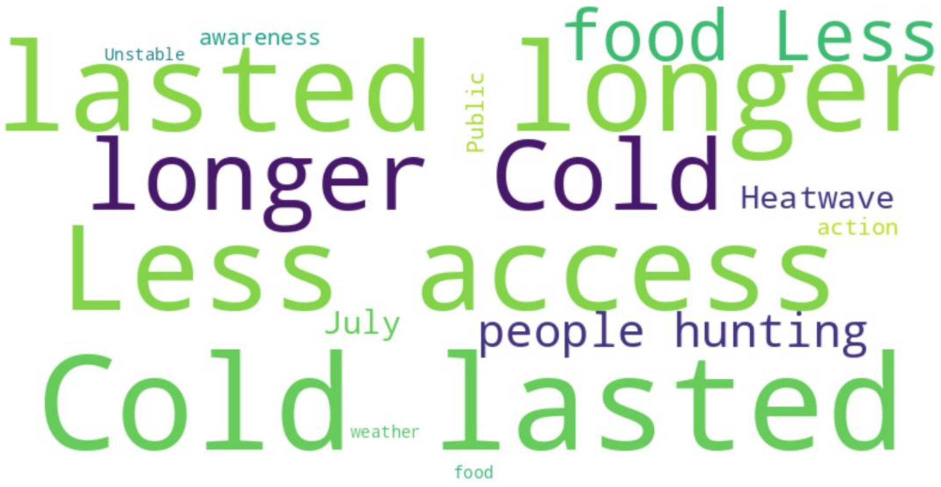

Appendix Figure A2. A word cloud illustrating the most frequently reported beekeeper perceptions regarding the decline in the hornet population in 2023.

A1.2 ***Survey Interpretation and Discussion***

The beekeeper survey conducted between 2022 and 2024 provides valuable insights into the perceived impact of *V. orientalis* on honey bee colonies in Malta. Across all three years, 100% of responding beekeepers reported sightings of *V. orientalis*, confirming its continued presence in Maltese apiaries. However, reported colony losses due to hornet predation fluctuated, with a peak in 2022 (63.8%), a decline in 2023 (29.4%), and a resurgence in 2024 (59.5%).

The pronounced decline in colony losses in 2023 appears to have been temporary. This may reflect variability in hornet population dynamics or external factors such as weather conditions, prey availability, or the effectiveness of implemented control measures. Beekeepers cited prolonged cold weather and limited food resources as potential contributors to reduced hornet activity in 2023 (**Appendix 1**). A colder-than-usual season may have delayed hornet colony establishment, reducing predation pressure [44]. Although queen trapping and manual hornet removal were reported as additional mitigating measures, a concurrent decrease in beekeeper intervention rates suggests that these efforts alone are unlikely to explain the trend. Shifts in urban food availability, such as increased access to alternative protein sources, may also have played a role in diverting hornet foraging away

from apiaries. These observations highlight the need for long-term ecological monitoring to better understand how environmental and anthropogenic factors shape *V. orientalis* activity and impact.

Reported intervention efforts declined over the three-year period, from 82.8% in 2022 to 73.5% in 2023 and 75.7% in 2024. Their perceived effectiveness also dropped, with 52.9% of respondents in 2023 reporting success compared to 43.2% in 2024. Simultaneously, the proportion of beekeepers reporting failed interventions increased from 11.8% to 18.9%. These trends suggest that traditional control strategies may be losing efficacy, underscoring the need to reassess current practices and explore more sustainable or integrated management approaches.

Honey bee losses were most frequently reported between July and October, corresponding to peak hornet predatory activity. This seasonal pattern likely reflects the increased protein demands of developing *V. orientalis* colonies, as worker hornets hunt insects such as honey bees to feed their larvae, while adults primarily consume carbohydrates like nectar and fruit. Microbiome findings related to foraging behaviour and dietary variation are explored in Discussion Section 4.3.

Appendix 2

Video are accessible at the following link: https://doi.org/10.17632/4ng7kx3nff.1

Video 1: **VID_20240916_083327.mp4**

Vespa orientalis hornets inside a Maltese honey bee colony. Video credit: Jorge Spiteri.

Video 2: **VID_20240929_164002.mp4**

Vespa orientalis hornets trapped at a Maltese apiary. Video credit: Jorge Spiteri.

Video 3: **VID_20240924_102707.mp4**

Apiary protection system from *Vespa orientalis* hornets. Video credit: Jorge Spiteri.

Appendix 3

This section is cited in Discussion Section 4.4.

Study Limitations

This study provides the first integrated analysis of the gut microbiota, predicted microbial functions, and pathogen carriage in *Vespa orientalis*, alongside a national beekeeper survey on its impact in Malta. While this multi-dimensional approach yielded novel ecological insights, several limitations should be acknowledged. First, there was no previously published data on the direct impact of *V. orientalis* on honey bee colonies, particularly within its native range. To address this gap, we incorporated beekeeper survey data to assess perceived predation patterns and colony losses. Second, the use of 16S rRNA gene amplicon sequencing constrained taxonomic resolution, particularly at the species or strain level. This limitation hampers the ability to distinguish pathogenic from commensal strains in key genera such as *Arsenophonus*, *Enterobacter*, and *Serratia*, and precludes functional validation of microbial roles in hornet health or pathogen dynamics. Third, the study design was cross-sectional and based on a single sampling period. As a result, it cannot account for seasonal shifts in hornet diet, microbial composition, or pathogen presence. Longitudinal sampling across different stages of colony development would be necessary to determine whether observed microbiota patterns are stable or influenced by seasonal or behavioural variability. Fourth, although bee-associated pathogens (*Nosema ceranae* and *Crithidia bombi*) were detected, we did not assess their viability or infectivity. Their presence alone does not confirm that hornets act as competent hosts or vectors. Future studies should test whether these pathogens survive gut passage, are shed in viable form, and contribute to environmental or pollinator-level transmission. Fifth, sampling was limited to worker females. Males, which do not forage for food and differ physiologically, were absent during the collection period. Including males in future work would help disentangle diet-driven from host-sex-driven microbiome differences. Sixth, sample sizes were uneven across sites, and one location (Qawra) had a particularly low number of individuals (n = 3). This disparity may reduce

statistical power and limit the generalisability of site-based comparisons, particularly regarding dietary and environmental influences. Furthermore, although compositional differences were observed between dietary groups and sampling sites, the ecological variability between sites and the limited sample size in certain locations constrain the strength of statistical comparisons. Controlled experiments, where dietary inputs are manipulated and stable, or comparative studies involving non-foraging males, would help confirm the influence of diet and geography more conclusively. Finally, although we hypothesised that pathogens may be acquired via prey ingestion, floral contamination, or environmental contact, these transmission routes were not experimentally tested. As this study was genomic and cross-sectional in design, further experimental validation, including floral exposure trials, gut viability assays, and controlled transmission studies, will be needed to confirm microbial acquisition and transmission mechanisms in *V. orientalis*.

To advance understanding of *V. orientalis* in pathogen ecology, future work should include seasonal and geographic replication, functional metagenomic profiling, and experimental testing of microbial viability and transmission. Such research would clarify whether *V. orientalis* acts as a transient microbial carrier, ecological sink, or potential participant in pathogen cycling within pollinator communities.
